# Posicionamento Brasileiro sobre Hipertensão Arterial Resistente – 2020

**DOI:** 10.36660/abc.20200198

**Published:** 2020-04-06

**Authors:** Juan Carlos Yugar-Toledo, Heitor Moreno, Miguel Gus, Guido Bernardo Aranha Rosito, Luiz César Nazário Scala, Elizabeth Silaid Muxfeldt, Alexandre Alessi, Andrea Araújo Brandão, Osni Moreira, Audes Diógenes de Magalhães Feitosa, Oswaldo Passarelli, Dilma do Socorro Moraes de Souza, Celso Amodeo, Weimar Kunz Sebba Barroso, Marco Antônio Mota Gomes, Annelise Machado Gomes de Paiva, Eduardo Costa Duarte Barbosa, Roberto Dischinger Miranda, José Fernando Vilela-Martin, Wilson Nadruz, Cibele Isaac Saad Rodrigues, Luciano Ferreira Drager, Luiz Aparecido Bortolotto, Fernanda Marciano Consolim-Colombo, Márcio Gonçalves de Sousa, Flávio Antonio de Oliveira Borelli, Sérgio Emanuel Kaiser, Gil Fernando Salles, Maria de Fátima de Azevedo, Lucélia Batista Neves Cunha Magalhães, Rui Manoel dos Santos Póvoa, Marcus Vinícius Bolívar Malachias, Armando da Rocha Nogueira, Paulo César Brandão Veiga Jardim, Thiago de Souza Veiga Jardim

**Affiliations:** 1 Faculdade Estadual de Medicina de São José do Rio Preto São José do Rio PretoSP Brasil Faculdade Estadual de Medicina de São José do Rio Preto, São José do Rio Preto, SP – Brasil; 2 Faculdade de Ciências Médicas Universidade Estadual de Campinas CampinasSP Brasil Faculdade de Ciências Médicas da Universidade Estadual de Campinas, Campinas, SP – Brasil; 3 Hospital Moinhos de Vento Porto AlegreRS Brasil Hospital Moinhos de Vento, Porto Alegre, RS – Brasil; 4 Universidade Federal de Ciências da Saúde de Porto Alegre Porto AlegreRS Brasil Universidade Federal de Ciências da Saúde de Porto Alegre, Porto Alegre, RS – Brasil; 5 Faculdade de Medicina Universidade Federal de Mato Grosso CuiabáMT Brasil Faculdade de Medicina da Universidade Federal de Mato Grosso, Cuiabá, MT – Brasil; 6 Hospital Universitário Clementino Fraga Filho Universidade Federal do Rio de Janeiro Rio de JaneiroRJ Brasil Hospital Universitário Clementino Fraga Filho, Universidade Federal do Rio de Janeiro, Rio de Janeiro, RJ – Brasil; 7 Universidade Federal do Paraná CuritibaPR Brasil Universidade Federal do Paraná, Curitiba, PR – Brasil; 8 Universidade do Estado do Rio de Janeiro Rio de JaneiroRJ Brasil Universidade do Estado do Rio de Janeiro, Rio de Janeiro, RJ – Brasil; 9 Pontifícia Universidade Católica do Paraná CuritibaPR Brasil Pontifícia Universidade Católica do Paraná, Curitiba, PR – Brasil; 10 Universidade Federal de Pernambuco RecifePE Brasil Universidade Federal de Pernambuco, Recife, PE – Brasil; 11 Instituto Dante Pazzanese de Cardiologia São PauloSP Brasil Instituto Dante Pazzanese de Cardiologia,São Paulo, SP – Brasil; 12 Faculdade de Medicina Universidade Federal do Pará BelémPA Brasil Faculdade de Medicina da Universidade Federal do Pará, Belém, PA – Brasil; 13 Faculdade de Medicina Universidade Federal de Goiás GoiâniaGO Brasil Faculdade de Medicina da Universidade Federal de Goiás, Goiânia, GO – Brasil; 14 Centro Universitário MaceióAL Brasil Centro Universitário CESMAC, Maceió, AL – Brasil; 15 Liga de Combate à Hipertensão de Porto Alegre Porto AlegreRS Brasil Liga de Combate à Hipertensão de Porto Alegre, Porto Alegre, RS – Brasil; 16 Universidade Federal de São Paulo São PauloSP Brasil Universidade Federal de São Paulo, São Paulo, SP – Brasil; 17 Faculdade de Ciências Médicas e da Saúde Pontifícia Universidade Católica de são Paulo São PauloSP Brasil Faculdade de Ciências Médicas e da Saúde Pontifícia Universidade Católica de são Paulo, São Paulo, SP – Brasil; 18 Hospital das Clínicas Faculdade Medicina Universidade de São Paulo São PauloSP Brasil Instituto do Coração do Hospital das Clínicas da Faculdade Medicina Universidade de São Paulo,São Paulo, SP – Brasil; 19 Universidade Federal do Rio Grande do Norte NatalRN Brasil Universidade Federal do Rio Grande do Norte, Natal, RN – Brasil; 20 Faculdade de Tecnologia e Ciências Universidade Federal da Bahia SalvadorBA Brasil Faculdade de Tecnologia e Ciências da Universidade Federal da Bahia, Salvador, BA – Brasil; 21 Faculdade de Ciências Médicas de Minas Gerais Belo horizonteMG Brasil Faculdade de Ciências Médicas de Minas Gerais, Belo horizonte, MG – Brasil; 22 Universidade Federal do Rio de Janeiro Rio de JaneiroRJ Brasil Universidade Federal do Rio de Janeiro, Rio de Janeiro, RJ – Brasil; 23 Faculdade de Medicina Universidade Federal de Goiás GoiâniaGO Brasil Faculdade de Medicina da Universidade Federal de Goiás, Goiânia, GO – Brasil; 24 Hospital do Coração de Goiás GoiâniaGO Brasil Hospital do Coração de Goiás, Goiânia, GO – Brasil

**Realização:** Departamento de Hipertensão Arterial (DHA) da Sociedade Brasileira de Cardiologia

**Conselho de Normatizações e Diretrizes (2020-2021):** Brivaldo Markman Filho, Antonio Carlos Sobral Sousa, Aurora Felice Castro Issa, Bruno Ramos Nascimento, Harry Correa Filho, Marcelo Luiz Campos Vieira

**Coordenador de Normatizações e Diretrizes (2020-2021):** Brivaldo Markman Filho

**Table:** 

Declaração de potencial conflito de interesses dos autores/colaboradores do Posicionamento Brasileiro sobre Hipertensão Arterial Se nos últimos 3 anos o autor/colaborador do Posicionamento:							
Nomes Integrantes do Posicionamento	Participou de estudos clínicos e/ou experimentais subvencionados pela indústria farmacêutica ou de equipamentos relacionados à diretriz em questão	Foi palestrante em eventos ou atividades patrocinadas pela indústria relacionados à diretriz em questão	Foi (é) membro do conselho consultivo ou diretivo da indústria farmacêutica ou de equipamentos	Participou de comitês normativos de estudos científicos patrocinados pela indústria	Recebeu auxílio pessoal ou institucional da indústria	Elaborou textos científicos em periódicos patrocinados pela indústria	Tem ações da indústria
Alexandre Alessi	Não	Não	Não	Não	Não	Não	Não
Andrea Araújo Brandão	Não	Abbott, EMS, Merck	Não	Não	Servier, Medley	Servier, Abbott	Não
Annelise Machado Gomes de Paiva	Não	Não	Não	Não	Não	Não	Não
Armando da Rocha Nogueira	Não	Não	Não	Não	Não	Não	Não
Audes Diógenes de Magalhães Feitosa	Não	Omron	Omron	Não	Não	Não	Não
Celso Amodeo	Medtronic	Não	Não	Não	Novo Nordisk	Não	Não
Cibele Isaac Saad Rodrigues	Não	Não	Não	Não	Não	Não	Não
Dilma do Socorro Moraes de Souza	Não	Não	Não	Não	Não	Não	Não
Eduardo Costa Duarte Barbosa	Não	Não	Não	Não	EMS, Servier	EMS, Novartis, Medley	Não
Elizabeth Silaid Muxfeldt	Não	Não	Não	Não	Não	Não	Não
Fernanda Marciano Consolim-Colombo	Não	Servier, Merck	Não	Não	Servier, Merck, Daiichi Sankyo	Servier, Merck	Não
Flavio Antonio de Oliveira Borelli	Não	Não	Não	Não	Não	Libbs	Não
Gil Fernando Salles	Não	Não	Não	Não	Não	Não	Não
Guido Bernardo Aranha Rosito	Não	Não	Não	Não	Não	Não	Não
Heitor Moreno Júnior	Não	Não	Não	Não	Não	Não	Não
José Fernando Vilela-Martin	Não	Não	Não	Não	Não	Não	Não
Juan Carlos Yugar-Toledo	Não	Não	Não	Não	Não	Não	Não
Luciano Ferreira Drager	Não	Não	Não	Não	Não	Não	Não
Lucélia Batista Neves Cunha Magalhães	Não	Não	Não	Não	Não	Não	Não
Luiz Aparecido Bortolotto	Não	Não	Não	Não	Não	Servier, Merck	Não
Luiz César Nazário Scala	Não	Não	Não	Não	Não	Não	Não
Márcio Gonçalves de Sousa	Não	Não	Não	Não	Não	Não	Não
Marco Antônio Mota Gomes	Não	Não	Servier, Torrent, Abbott, Omron, Novartis, Astrazeneca	Não	Servier, Torrent, Abbott, Omron, Novartis, Astrazeneca	Servier, Torrent, Abbott, Omron, Novartis, Astrazeneca	Não
Marcus Vinícius Bolívar Malachias	Não	Abbott, Biolab, Libbs, Novo Nordisk, Takeda	Não	Não	Não	Abbott, Biolab, Farmoquímica, Libbs, Novo Nordisk	Não
Maria de Fátima de Azevedo	Não	Não	Não	Não	Não	Não	Não
Miguel Gus	Não	Não	Não	Não	Não	Não	Não
Osni Moreira Filho	Não	Servier, Merck	Não	Não	Não	Não	Não
Oswaldo Passarelli Júnior	Não	Não	Não	Não	Não	Não	Não
Paulo César Brandão Veiga Jardim	Não	Não	Não	Não	Não	Biolab, Aché, Libbs	Não
Roberto Dischinger Miranda	Não	Não	Não	Não	Não	Não	Não
Rui Manoel dos Santos Póvoa	Não	Não	Não	Não	Não	Não	Não
Sérgio Emanuel Kaiser	Não	Não	Não	Não	Não	Novartis	Não
Thiago de Souza Veiga Jardim	Não	Torrent	Não	Não	Libbs, Torrent, Novartis	Torrent	Não
Weimar Kunz Sebba Barroso	Amgen, AstraZeneca, Torrent, EMS, Novartis	Não	Não	Não	EMS, Sandoz, Servier, Novartis	Medley, Sandoz, EMS	Não
Wilson Nadruz Júnior	Não	Não	Não	Não	Não	Não	Não

Sumário

1. Definição e Epidemiologia 580

1.1. Definição/Novos Conceitos 580

1.2. Controle da Hipertensão Arterial no Brasil e no Mundo 580

1.3. Incidência e Prevalência de Hipertensão Arterial Resistente 580

1.4. Fatores Relacionados à Hipertensão Arterial Resistente 580

2. Aspectos Prognósticos 581

2.1. Introdução 581

2.2. Pressão Arterial de Consultório e Monitoramento Ambulatorial da Pressão Arterial 581

2.3. Lesões de Órgãos-Alvo 581

**2.3.1. Pressão Arterial Central e Rigidez Arterial 581**

**2.3.2. Hipertrofia Ventricular Esquerda 581**

**2.3.3. Albuminúria 581**

**2.3.4. Biomarcadores Inflamatórios 581**

3. Fluxograma de Avaliação de Hipertensão Arterial Resistente 581

3.1. Fluxograma na Abordagem Diagnóstica da Hipertensão Arterial Resistente 581

4. Medida da Pressão Arterial 582

4.1. Pressão Arterial de Consultório na Hipertensão Arterial Resistente 582

4.2. Monitoramento Ambulatorial da Pressão Arterial em Hipertensão Arterial Resistente 582

4.3. Monitoramento Residencial da Pressão Arterial e Automedida da Pressão Arterial 583

4.4. Medida da Pressão Arterial Central 583

5. Lesões de Órgãos-Alvo 583

5.1. Introdução 583

5.2. Alterações Vasculares 583

5.3. Alterações Cerebrais 583

5.4. Alterações Cardíacas 584

5.5. Alterações Renais 584

6. Fenótipo do Paciente com Hipertensão Arterial Resistente 585

6.1. Introdução 585

6.2. Fenótipo do Paciente com Hipertensão Arterial Resistente 585

6.3. Fenótipo da Hipertensão Arterial Resistente Controlada e da Não Controlada 585

**6.3.1. Aspectos Fisiopatológicos 585**

**6.3.2. Diferenças Clínicas 585**

**6.3.3. Prognóstico 585**

6.4. Fenótipo do Paciente com Hipertensão Arterial Refratária 585

7. Causas Secundárias de Hipertensão Arterial Resistente 586

7.1. Introdução 586

7.2. Hipertensão Arterial Secundária de Causas Não Endócrinas 586

**7.2.1. Apneia Obstrutiva do Sono 586**

**7.2.2. Doença do Parênquima Renal 586**

**7.2.3. Estenose da Artéria Renal 587**

7.3. Hipertensão Arterial Secundária de Causas Endócrinas 587

**7.3.1. Hiperaldosteronismo Primário 587**

**7.3.2. Feocromocitoma 587**

**7.3.3. Hipotireoidismo e Hipertireoidismo 588**

8. Tratamento Não Farmacológico 589

8.1. Perda Ponderal 589

8.2. Restrição de Sal 589

8.3. Ingestão de Álcool 589

8.4. Atividade Física 589

9. Tratamento Farmacológico da Hipertensão Arterial Resistente) 589

10. Novos Tratamentos da Hipertensão Arterial Resistente 590

10.1. Introdução 590

10.2. Estimulação Direta do Seio Carotídeo 590

10.3. Denervação Simpática Renal 591

10.4. Uso de Pressão Positiva Contínua em Vias Aéreas 591

10.5. Fístula Arteriovenosa 591

Referências 591

## 1. Definição e Epidemiologia

**Coordenador:** Heitor Moreno Júnior.

**Autores:** Juan Carlos Yugar-Toledo, Heitor Moreno Júnior, Miguel Gus, Guido Bernardo Aranha Rosito e Luiz César Nazário Scala.

### 1.1. Definição/Novos Conceitos

A hipertensão arterial resistente (HAR) é definida quando a pressão arterial (PA) permanece acima das metas recomendadas com o uso de três anti-hipertensivos de diferentes classes, incluindo um bloqueador do sistema renina-angiotensina (inibidor da enzima conversora da angiotensina [IECA] ou bloqueador do receptor de angiotensina [BRA]), um bloqueador dos canais de cálcio (BCC) de ação prolongada e um diurético tiazídico (DT) de longa ação em doses máximas preconizadas e toleradas, administradas com frequência, dosagem apropriada e comprovada adesão.

Outros fármacos podem ser associados aos primeiros em caso de falha deles (antagonistas da aldosterona, betabloqueadores e α-metildopa); entretanto, especialistas conflitam sobre o assunto em alguns pontos referentes à dose/potência, embora a maior discussão seja quanto ao uso da clortalidona ou da hidroclorotiazida como principais DT.^1^

Nesta definição está incluído o subgrupo de pacientes hipertensos resistentes, cuja PA é controlada com quatro ou mais medicamentos anti-hipertensivos, chamada de HAR controlada (HAR-C).^2 , 3^ A classificação da doença em HAR-C e HAR não controlada (HAR-NC),^4^ incluindo a HAR refratária (HAR-Ref), um fenótipo extremo de HAR-NC em uso de cinco ou mais anti-hipertensivos,^5^ é uma proposta que ganha espaço na literatura.^6 , 7^

Assim, HAR-NC é definida como uma PA que permanece acima do nível desejado (140/90 mmHg), apesar do uso concomitante de quatro ou mais agentes anti-hipertensivos de diferentes classes e um quarto fármaco, que geralmente é um antagonista do receptor mineralocorticoide ou um bloqueador simpático central ( Quadro 1 ).

**Quadro 1:** – Classificação da hipertensão arterial resistente

	Número de anti-hipertensivos	
HIpertensão resistente controlada	N	Hipertensão resistente não controlada
	6	
	5	
	4	
	3	Hipertensão resistente
	2	
	1	
< 140/90	Pressão arterial (mmHg)	≥ 140/90
Normotensão		Hipertensão

### 1.2. Controle da Hipertensão Arterial no Brasil e no Mundo

A análise de 135 estudos populacionais com 1 milhão de indivíduos indica que 31,1% da população adulta é hipertensa (IC 95%; 30 a 32%), com valor estimado em 28,5 e 31,5% nos países de maior e menor condição socioeconômica, respectivamente. O controle pressórico varia dependendo das condições socioeconômicas, chegando a 28,4% nos países mais desenvolvidos e apenas 7,7% naqueles com menor grau de desenvolvimento.^8^No Brasil, a taxa de controle varia de 10,4 a 35,2% nas populações estudadas em três regiões do país.^9^

Um estudo envolvendo 291 centros das cinco regiões brasileiras e 2.810 pacientes avaliou a taxa de controle conforme perfil de risco e metas pressóricas. Para pacientes de menor risco e meta < 140/90 mmHg, o controle foi de 61,7%, enquanto para hipertensos de alto risco com meta < 130/80 mmHg o valor correspondente foi de 41,8%.^10^

### 1.3. Incidência e Prevalência de Hipertensão Arterial Resistente

A prevalência de HAR no mundo é estimada entre 10 e 20% dos hipertensos, o que significa aproximadamente 200 milhões de hipertensos resistentes.^11^ A variabilidade deve-se, principalmente, à diferença de critérios para HAR e às características das populações estudadas.

O National Health and Nutrition Examination Survey (NHANES) demonstrou uma prevalência de cerca de 9% dos hipertensos tendo HAR, correspondendo a 12,8% daqueles que utilizam anti-hipertensivos nos EUA.^12^

No entanto, a real prevalência de HAR não é conhecida. Uma metanálise de Achelrod et al.^11^avaliando populações de hipertensos tratados encontrou uma prevalência de 13,72% (IC 95%; 11,19 a 16,24%), de acordo com 20 estudos observacionais, e 16,32% (IC 95%; 10,68 a 21,95%) para quatro ensaios clínicos randomizados.^11^No Brasil, um estudo multicêntrico e utilizando o monitoramento ambulatorial da pressão arterial (MAPA) (ReHOT study) mostrou uma prevalência de HAR de 11,7%.^13^

Daugherty et al.^14^ analisaram a incidência de HAR em 205.750 hipertensos que iniciaram tratamento anti-hipertensivo entre 2002 e 2006. A taxa foi de 1,9% com um ano e meio de acompanhamento (0,7 por 100 pacientes ao ano), acarretando um risco cardiovascular (CV) 1,47 maior em 3,8 anos.^14^

### 1.4. Fatores Relacionados à Hipertensão Arterial Resistente

A HAR é mais prevalente em idosos, obesos e afrodescendentes, bem como em pacientes com hipertrofia ventricular esquerda, diabetes melito, nefropatia crônica, síndrome metabólica, elevada ingestão de álcool e/ou sal e sedentarismo.^1 , 15 - 17^ Os fatores relacionados à HAR abrangem inúmeros aspectos, como: 1) diagnósticos: técnica inadequada de aferição da PA, efeito do avental branco;^1 , 15^2) causais: maior sensibilidade ao sal, expansão volêmica por ingestão excessiva de sal ou doença renal crônica (DRC), uso de anti-inflamatórios não hormonais, esteroides anabólicos, contraceptivos orais, simpaticomiméticos (descongestionantes nasais, inibidores do apetite, cocaína), quimioterápicos, antidepressivos, eritropoietina, imunodepressores, álcool;^1 , 15^ 3) causas secundárias de hipertensão, destacando-se: hiperaldosteronismo primário, apneia obstrutiva do sono (AOS), DRC, estenose de artéria renal, doenças tireóideas;^15^ 4) terapêuticos: fármacos inapropriados ou em doses insuficientes, inércia médica, baixa aderência.^16 , 17^ Tanto a hipertensão sistólica quanto a diastólica podem ser resistentes, sendo a primeira mais prevalente.^1^

## 2. Aspectos Prognósticos

**Coordenadora:** Elizabeth Silaid Muxfeldt.

**Autores:** Alexandre Alessi, Andrea Araújo Brandão, Osni Moreira Filho e Elizabeth Silaid Muxfeldt.

### 2.1. Introdução

A HAR está relacionada a uma alta morbimortalidade CV, apresentando um risco 47% maior de desenvolver eventos CV quando comparados aos hipertensos em geral.^14^

### 2.2. Pressão Arterial de Consultório e Monitoramento Ambulatorial da Pressão Arterial

A HAR verdadeira, diagnosticada pelo MAPA, tem o dobro do risco CV em comparação à HAR relacionada a efeito do avental branco.^18^ De modo geral, as médias de PA obtidas nos três períodos do MAPA são fortes preditoras de risco CV, enquanto a PA de consultório não demonstrou nenhum valor prognóstico.^18 , 19^Estudos longitudinais destacaram a PA no sono elevada e a ausência de descenso noturno como importantes preditores de risco CV.^18 - 20^ A importância prognóstica do comportamento noturno da PA também já foi demonstrada em metanálises.^21^

### 2.3. Lesões de Órgãos-Alvo

#### 2.3.1. Pressão Arterial Central e Rigidez Arterial

A velocidade de onda de pulso (VOP) tem valor preditivo independente em vários subgrupos de pacientes hipertensos.^22^ Nos resistentes, foi observado maior rigidez arterial que nos controlados, sendo um marcador de prognóstico e de resposta terapêutica anti-hipertensiva.^23^ Em hipertensos existe um valor aditivo quando a VOP é agregada a escores de risco CV.^24^

#### 2.3.2. Hipertrofia Ventricular Esquerda

O diagnóstico eletrocardiográfico de hipertrofia ventricular esquerda (HVE) foi preditor de risco para doença coronariana (índice de Cornell) e cerebrovascular (índice de Sokolow-Lyon), e a regressão desses dois índices reduziu o risco de eventos CV em 35 e 40%, respectivamente.^25^

#### 2.3.3. Albuminúria

Na HAR há implicações prognósticas tanto da albuminúria inicial como do seu padrão evolutivo. Em uma grande coorte prospectiva com 531 hipertensos resistentes, a albuminúria moderadamente elevada (AME) inicial foi um preditor independente de eventos compostos e mortalidade total.^26^ Uma nova análise do mesmo grupo, porém envolvendo 1.048 pacientes, identificou que a AME aumenta em 40% o risco de eventos CV fatais e não fatais e de mortalidade total.^27^

Evolutivamente, a persistência da AME em 2 anos foi um fator de risco para eventos CV, enquanto a normoalbuminúria persistente foi fator de proteção.^26^ Outra coorte envolvendo 143 pacientes com HAR, analisados no momento basal e após 6 anos de seguimento, mostrou que o desenvolvimento de AME ou a sua persistência estavam relacionados a maior risco de eventos CV. Em contrapartida, a persistência de normoalbuminúria, ou a regressão da AME, se associou a menor risco de eventos maiores.^28^

#### 2.3.4. Biomarcadores Inflamatórios

A proteína C reativa elevada foi preditora independente de doença coronariana e cerebrovascular, sendo um marcador mais importante para hipertensos resistentes mais jovens, obesos, com MAPA não controlado e padrão não *dipper* (descenso noturno ausente ou atenuado).^29^

## 3. Fluxograma de Avaliação de Hipertensão Arterial Resistente

**Coordenador:** Audes Diógenes de Magalhães Feitosa.

**Autores:** Oswaldo Passarelli Júnior, Dilma do Socorro Moraes de Souza e Audes Diógenes de Magalhães Feitosa.

### 3.1. Fluxograma na Abordagem Diagnóstica da Hipertensão Arterial Resistente

**Figura 1:**
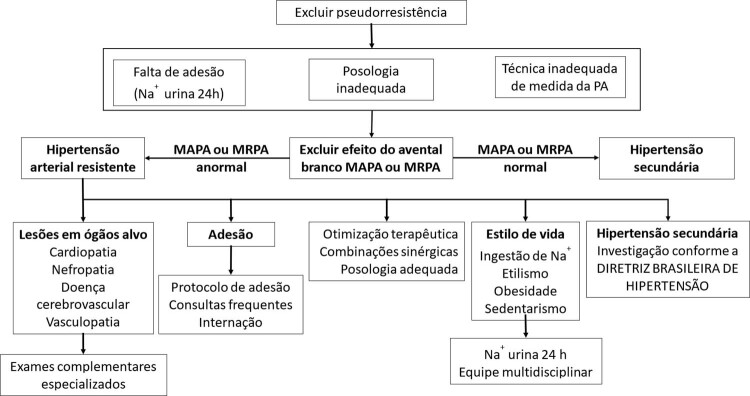
***–** Fluxograma de avaliação da hipertensão arterial resistente. MAPA: monitoramento ambulatorial da pressão arterial; MRPA: monitoramento residencial da pressão arterial; Na+: sódio; PA: pressão arterial.*

Diante da suspeita clínica de HAR, é necessário verificar a confirmação diagnóstica, e a primeira etapa na investigação é a exclusão das causas de pseudorresistência, tais como falta de adesão ao tratamento (farmacológico e não farmacológico), posologia inadequada, técnica imprópria de aferição da PA e efeito do avental branco^1^ ( Figura 1 ). O MAPA e o monitoramento residencial da pressão arterial (MRPA) são os exames para confirmação do controle inadequado da PA.^30 - 32^

Uma vez afastada a pseudorresistência, confirma-se a existência da HAR e inicia-se uma investigação diagnóstica com exames específicos, conforme a orientação das Diretrizes de Hipertensão em relação ao comprometimento de lesões em órgãos-alvo e hipertensão secundária.^33 , 34^A ocorrência de comorbidades associadas deve ser detectada com exames especializados de acordo com a suspeita clínica.

A medida da PA fora do consultório é fundamental, pois ela costuma ser mais elevada em relação à medida domiciliar; desse modo, o efeito do avental branco é frequente nessa população. A adesão ao tratamento é sempre um grande desafio, especialmente nos serviços públicos.

Alguns problemas relacionados aos pacientes podem ocorrer, como: rejeição a número excessivo de fármacos em posologias complexas (muitas tomadas e comprimidos), efeitos colaterais dos medicamentos, problemas socioculturais e desconhecimento da história natural da doença, além de outros referentes ao médico, como: relação médico-paciente ruim, posologias não sinérgicas ou doses equivocadas e omissão ou desconhecimento na investigação das causas secundárias tratáveis. Um problema relacionado aos serviços de saúde pode ser a dificuldade de acesso aos médicos, medicamentos e exames complementares.

Todos esses fatores dificultam a adesão ao tratamento farmacológico e não farmacológico; por isso, devem ser verificados e contornados.

A ingesta de sal precisa sempre ser conferida, se possível com a verificação do sódio em urina de 24 horas, pois frequentemente a ingestão é excessiva em função do consumo de alimentos industrializados e do desconhecimento dos pacientes em relação ao consumo excessivo de sal.

A otimização terapêutica deve ser realizada preferencialmente com o mesmo médico, por um período mínimo de 6 meses, para o fortalecimento da relação médico-paciente. Somam-se a isso orientação constante em relação ao estilo de vida saudável e a verificação contínua da adesão ao tratamento, com esquemas posológicos sinérgicos e ajustes medicamentosos adequados, respeitando a existência de comorbidades que indiquem ou contraindiquem determinada classe de fármaco anti-hipertensivo.

## 4. Medida da Pressão Arterial

**Coordenador:** Celso Amodeo.

**Autores:** Weimar Kunz Sebba Barroso, Marco Antônio Mota Gomes, Annelise Machado Gomes de Paiva e Eduardo Costa Duarte Barbosa.

### 4.1. Pressão Arterial de Consultório na Hipertensão Arterial Resistente

A verificação da PA de consultório, apesar de não ser diagnóstica para HAR, deve ser realizada, e o procedimento de aferição precisa seguir as orientações da VII Diretriz Brasileira de Hipertensão.^33^ A verificação da PA pode ser feita com esfigmomanômetros manuais, semiautomáticos ou automáticos. A recomendação é realizá-la várias vezes com o paciente sentado em ambiente calmo e confortável, para melhorar a reprodutibilidade e aproximar os valores obtidos no consultório àqueles fornecidos pelo MAPA na vigília.

Deve ser observada a possibilidade de ocorrer efeito do avental branco, fenômeno que envolve duas situações. A primeira é a hipertensão do avental branco, quando a PA está elevada em medidas isoladas no consultório e normais no MAPA ou no MRPA. A segunda situação é o efeito do avental branco, caracterizado por PA de consultório elevada em relação à média de pressão de vigília no MAPA ou à média semanal do MRPA, sem haver alteração do diagnóstico, seja de hipertensão ou normotensão.^35^

Essas duas situações podem levar ao falso diagnóstico de HAR, acarretando a realização de exames e o uso de medicação de modo desnecessário. É possível referir-se à hipertensão do avental branco como uma causa de hipertensão arterial pseudorresistente.^36^

### 4.2. Monitoramento Ambulatorial da Pressão Arterial em Hipertensão Arterial Resistente

Esse exame é necessário para afastar a hipótese de hipertensão do avental branco, que falsamente sugere HAR.^37^ O diagnóstico se confirma quando as médias pressóricas nos períodos de vigília e de 24 h estão abaixo de 135/85 mmHg e de 130/80 mmHg, respectivamente. Quando comparados com as medidas casuais da PA, os valores obtidos são mais fortemente relacionados com os riscos decorrentes da hipertensão arterial, principalmente nos exames de MAPA nos quais se identifica uma ausência ou atenuação do descenso da pressão com o sono, como também um aumento no diferencial de pressão sistólica-diastólica.^37^ O Quadro 2 apresenta as principais aplicabilidades do MAPA em hipertensão arterial, exame fundamental na avaliação, no diagnóstico e na evolução da HAR.

**Quadro 2:** – Principais informações obtidas com o monitoramento ambulatorial da pressão arterial

Múltiplas medidas ao longo de um período de observação
Avaliação pressórica durante o período de vigília
Correlação das medidas de vigília com atividades e sintomas
Avaliação pressórica durante o sono
Possibilidade de correlação da variabilidade pressórica com sintomas, atividades e fármacos
Complementação diagnóstica e prognóstica do paciente
Avaliação do efeito anti-hipertensivo

### 4.3. Monitoramento Residencial da Pressão Arterial e Automedida da Pressão Arterial

As medidas domiciliares da PA são mais precisas do que a medida casual e apresentam melhor predição de risco para os desfechos CV, contribuindo para maior adesão ao tratamento medicamentoso.^35 , 38 , 39^ Nesse contexto, a MRPA e a automedida da PA (AMPA) se apresentam como alternativas viáveis e eficazes tanto para o diagnóstico adequado quanto para a melhora na adesão.^40 , 41^

### 4.4. Medida da Pressão Arterial Central

A rigidez arterial é reconhecida como um importante índice prognóstico e potencial alvo terapêutico em pacientes hipertensos. Em função disso, a pressão sistólica central (PSc) e a VOP foram investigadas recentemente em uma população de pacientes com HAR.^42^ A idade média dessa população era de 58,7 ± 15,3 anos, e 65% (n = 53) eram do sexo feminino. A pressão braquial e central estavam aumentadas em todos os pacientes, e o valor de VOP foi superior ao valor de referência para a idade, sendo essa diferença estatisticamente maior para VOP no sexo feminino.

Outro estudo^23^ tratou de observar se existia uma associação entre HAR e rigidez arterial e mostrou que os pacientes com HAR apresentaram maior rigidez vascular do que o grupo com hipertensão bem controlada. A VOP aumentou com a rigidez arterial e foi correlacionada aos níveis de PA, justificando a necessidade de um controle adequado dela.

## 5. Lesões de Órgãos-Alvo

**Coordenador:** Roberto Dischinger Miranda.

**Autores:** José Fernando Vilela-Martin, Juan Carlos Yugar-Toledo, Wilson Nadruz Júnior e Cibele Isaac Saad Rodrigues.

### 5.1. Introdução

A HAR, controlada ou não, está associada a maior prevalência de lesão de órgãos-alvo (LOA) e a maior risco CV e de mortalidade, quando comparada à hipertensão arterial controlada.^43 - 45^ Por isso, a investigação de LOA na HAR é fundamental para complementar a estratificação de risco e estabelecer o prognóstico.^44^

### 5.2. Alterações Vasculares

Hipertensos resistentes apresentam alterações vasculares funcionais e estruturais decorrentes não só da hipertensão arterial não controlada, mas também do envelhecimento vascular precoce.. Esse é um processo complexo que envolve alterações bioquímicas, enzimáticas e celulares que modificam a função e a estrutura do vaso, culminando com a degeneração precoce e progressiva da saúde arterial.^43 - 47^

Os mecanismos fisiopatológicos incluem aumento do estresse oxidativo, disfunção endotelial, remodelamento vascular, hipertrofia de células musculares lisas, aumento da rigidez arterial por alterações na distribuição de colágeno/elastina, inflamação vascular e maior expressão de mediadores inflamatórios e metaloproteinases de reparação de matriz, além de aumento dos produtos finais da glicação avançada e calcificação parietal.^48 , 49^

Entre os mecanismos moleculares do envelhecimento vascular, citam-se as alterações genéticas de segmentos envolvidos na proteção e reparação do DNA^50^ e na atividade metabólica mitocondrial.^51^

Na microcirculação, a disfunção endotelial promove vasoconstrição, remodelamento eutrófico (aumento da relação média/lúmen [M/L] sem modificação externa), diminuição da reserva vasodilatadora e rarefação vascular, esta última avaliada por capilaroscopia *in vivo* ,^52^ biópsia de glúteo ou, ainda, por mensuração da relação M/L com Dopplerfluxometria a *laser* de artérias retinianas^53^ e videomicroscopia óptica. Nas grandes artérias, o remodelamento parietal leva ao aumento da rigidez arterial.^49 , 54 - 56^

A rigidez arterial é estimada pela VOP carótida-femoral (VOP c-f), e o cálculo do índice de incremento ( *augmentation index (AIx)* , por tonometria de aplanação.^57 , 58^ Essas alterações de parâmetros hemodinâmicos e biomarcadores celulares da rigidez arterial estão associadas ao aumento da morbimortalidade.^59 , 60^

O comprometimento macrovascular é caracterizado ainda pelas doenças aterosclerótica carotídea, cerebral, coronariana e periférica.^61 , 62^

### 5.3. Alterações Cerebrais

O comprometimento cerebrovascular na HAR ocorre de maneira sutil e insidiosa. Lesões microscópicas da substância branca têm início precoce e podem evoluir de modo irreversível, promovendo déficit cognitivo e progressão para demência vascular.^63 , 64^

Pacientes com HAR têm maior risco de infarto cerebral e isquemia cerebral transitória, fato apontado pelos estudos KAISER Permanente^16^ e REGARDS,^65^ que mostram um aumento de risco de 17 e 14%, respectivamente. Aterosclerose carotídea e dos pequenos vasos cerebrais são responsáveis por fenômenos isquêmicos e tromboembólicos. A oclusão de artéria retiniana é um marcador de lesão de pequenos vasos e tem sido associada a maior risco de evento cerebral.^66^

Hipertensão não controlada é a principal causa de acidente vascular hemorrágico. Pacientes com HAR apresentam microangiopatia (aneurismas de Charcot-Bouchard), que afetam as artérias penetrantes do cérebro e causam hemorragia intraparenquimatosa.^67^

Alterações na rigidez de grandes artérias também estão associadas a maior ocorrência de alterações microvasculares e maior predisposição para eventos cerebrovasculares.^68^

### 5.4. Alterações Cardíacas

Diversas alterações cardíacas, tais como HVE, disfunção diastólica do ventrículo esquerdo (DDVE) e isquemia miocárdica, podem ser observadas em pacientes com HAR.^69^ A HVE é um preditor independente de insuficiência cardíaca, doença arterial coronária (DAC), arritmias e acidente vascular encefálico.^70^

No Brasil, a prevalência de HVE em pacientes com HAR, avaliada por ecocardiografia, varia de 68 a 87%,^71 , 72^ sendo a HVE concêntrica ao padrão geométrico mais encontrado nesses indivíduos.^72 , 73^

A DDVE predispõe a eventos cardiovasculares e insuficiência cardíaca, independentemente da massa cardíaca e dos níveis de PA.^74^ A prevalência exata de DDVE em pacientes com HAR é incerta, mas a forte associação entre essa condição e a HVE^74^sugere que a DDVE é bastante frequente nessa população. Cerca de um terço dos pacientes com HAR têm diagnóstico de DAC.^71^ Contudo, mesmo na ausência de DAC manifesta, até 28% dos pacientes com HAR apresentam isquemia miocárdica,^72^ a qual pode resultar de diminuições na reserva coronária, de aumentos no consumo de oxigênio miocárdico, especialmente nos portadores de HVE, e de aumentos na rigidez arterial.^70 , 74^

### 5.5. Alterações Renais

A associação entre HAR e DRC está bem estabelecida, podendo ser causa ou consequência. O substrato anatomopatológico é a nefroesclerose hipertensiva, decorrente de alterações hemodinâmicas (hiperfiltração e hipertrofia glomerular) que culminam em glomeruloesclerose. A nefroesclerose, denominada erroneamente como “benigna”, caracteriza-se por arteriosclerose e arteriolosclerose, hialinose, lesões tubulointersticiais e glomerulosclerose segmentar focal e global.

São fatores de risco conhecidos para progressão da DRC: idade > 50 anos, sexo masculino, predisposição genética, história familiar, afrodescendência, duração e estágio da hipertensão arterial, baixo nível socioeconômico, intensidade da albuminúria, grau de disfunção renal, dislipidemia, obesidade, diabetes, estilo de vida (dieta hipersódica, hiperproteica e fumo), uso de substâncias nefrotóxicas, entre outros.^75^ Albuminúria e redução do ritmo de filtração glomerular estimado (RFG-e) identificam pacientes de alto risco CV e renal, e a diminuição da albuminúria pode ser objetivo terapêutico na HAR.^26 - 28^

**Figura 2:**
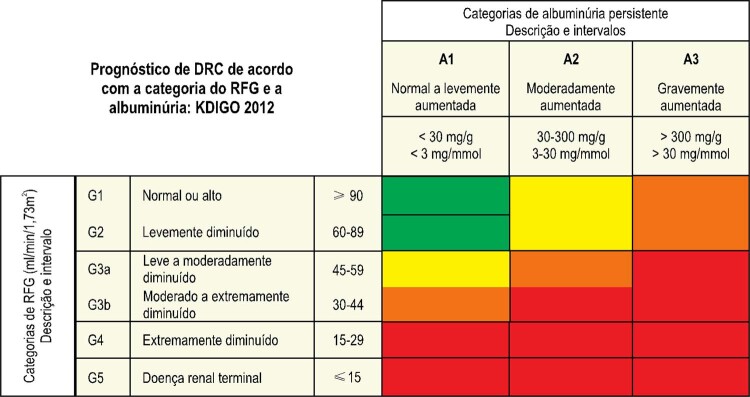
***–** Prognóstico da doença renal crônica de acordo com os graus de albuminúria e de declínio do RFG-e.^76^ Verde: baixo risco; amarelo: risco moderado; laranja: alto risco; vermelho: risco muito alto.*

Na avaliação e no acompanhamento da lesão renal, são recomendados: exame de urina, creatinina sérica para estimar o RFG pelas fórmulas MDRD ou CKD-EPI, disponíveis no site http://ckdepi.org/equations/gfr-calculator/, ultrassonografia renal e de vias urinárias e cálculo da razão albuminúria ou proteinúria/creatininúria visando à classificação do estágio de DRC^75^ ( Figura 2 ).

## 6. Fenótipo do paciente com Hipertensão Arterial Resistente

**Coordenador:** Luciano Ferreira Drager

**Autores:** Heitor Moreno Júnior, Juan Carlos Yugar-Toledo e Luiz Aparecido Bortolotto

### 6.1. Introdução

Nesta seção, serão descritas inicialmente as características que distinguem um hipertenso resistente de um não resistente. Posteriormente, serão discutidas as diferenças entre os hipertensos resistentes controlados e os não controlados, finalizando com a abordagem do fenótipo extremo dos pacientes com hipertensão resistente, que é o hipertenso refratário.

### 6.2. Fenótipo do paciente com Hipertensão Arterial Resistente

O paciente com HAR apresenta comumente algumas características que o distinguem daqueles com a não resistente, tais como: idade mais avançada; obesidade; perfil de alta ingestão de sal; DRC; diabetes; presença de LOA, como a hipertrofia ventricular esquerda; sexo feminino e raça negra.^1^ O estudo multicêntrico brasileiro ReHOT mostrou que diabetes, história prévia de acidente vascular encefálico e PA na entrada do estudo ≥ 180/110 mmHg (estágio 3 da hipertensão arterial) foram preditores independentes da verdadeira resistência.^13^ Enquanto algumas dessas características são intuitivas, outras como ser do sexo feminino, ainda não têm explicações bem definidas para a predição na HAR.

### 6.3. Fenótipo da Hipertensão Arterial Resistente Controlada e da Não Controlada

#### 6.3.1. Aspectos Fisiopatológicos

A HAR-C evidencia maior dependência do *status* volêmico que a HAR-NC, devido a importante persistência de retenção hídrica, sensibilidade aumentada ao sódio, hiperaldosteronismo e disfunção renal. Além disso, maior expansão de conteúdo plasmático avaliado por bioimpedância torácica,^77^ maior concentração de aldosterona plasmática e urinária, supressão da atividade de renina,^78^ elevada relação aldosterona/renina plasmática, assim como altos níveis de peptídeo natriurético atrial (ANP) e cerebral (BNP) são observados nesses indivíduos.^79 - 83^ Essa relação entre volume e pressão elevados é a base fisiopatológica demonstrada em vários estudos^81 , 84 , 85^e justifica o uso de diuréticos em pacientes com HAR-C.^86 , 87^

Em contraste, portadores de HAR-NC frequentemente têm hiperatividade do sistema simpático, evidenciada por elevação de metanefrinas urinárias (24 h) e da frequência cardíaca de repouso e redução da sua variabilidade em 24 h (análise espectral), além de maior rigidez vascular (aumento da VOP).^88 , 89^ Esses marcadores de atividade simpática aumentada, em conjunto com outros fatores ligados ao hiperaldosteronismo,^78 , 90 - 92^ estão vinculados a mecanismos que mantêm a PA alta mesmo com o uso de quatro ou mais agentes anti-hipertensivos, caracterizando a HAR-NC. Valores mais elevados de VOP denotam rigidez arterial exacerbada,^4^ e níveis elevados de citocinas, incluindo o fator de necrose tumoral alfa (TNF-α),^48 , 56 , 93^ provavelmente assinalam o dano vascular em pacientes com HAR.^49^

Outros fatores e mecanismos, como idade, obesidade, AOS,^4 , 94 , 95^ afrodescendência, adipocitocinas alteradas,^96^ disfunção endotelial, maior atividade das metaloproteinases-2 e 9 e das moléculas de adesão^97 - 99^ também estão envolvidos nesse processo.

**Figura 3:**
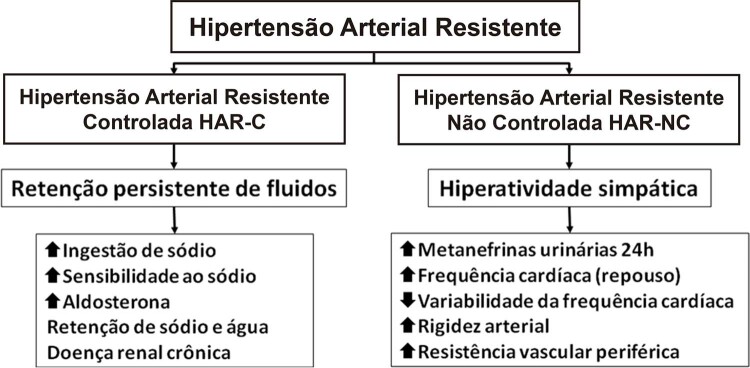
***–** Mecanismos fisiopatológicos predominantes na hipertensão resistente controlada (HAR-C) e não controlada (HAR-NC). Hipertensão arterial refratária (não controlada com cinco fármacos ou mais) é incluída no grupo da HAR-NC.*

Polimorfismos genéticos, especialmente do sistema renina-angiotensina-aldosterona e da sintase endotelial do óxido nítrico (eNOS) vêm sendo correlacionados à HAR^100 , 101^ ( Figura 3 ); todavia, grandes estudos convenientemente caracterizados em indivíduos com a doença são necessários para definir a importância da genética nesse grupo de pacientes.

#### 6.3.2. Diferenças Clínicas

Em 2011, Martins et al. publicaram um estudo comparativo entre pacientes com HAR-C e HAR-NC,^4^ mais exatamente sobre fatores biológicos que contribuiriam para a resistência aos anti-hipertensivos. Os índices de massa corporal, rigidez arterial (VOP), índice de massa ventricular esquerda (IMVE) e concentração de aldosterona plasmática (AP) foram maiores no grupo da HAR-NC, quando comparados com o grupo da HAR-C. Além disso, por análise multivariada, os autores demonstraram que a VOP era dependente da idade nos grupos, embora sua influência fosse mais importante nos pacientes com HAR-NC.

Eles também demonstraram que o grupo da HAR-NC apresentava maiores valores de espessura íntima média de carótidas (EIMC) e VOP.^102^ Finalmente, o descenso do sono ( *dipping pattern* ) foi menos pronunciado no grupo da HAR-NC.^103^

#### 6.3.3. Prognóstico

Pierdomenico et al.^104^ avaliaram desfechos CV em indivíduos com HAR-C e HAR-NC. A ocorrência de eventos CV fatais e não fatais foi investigada em 340 pacientes com HAR-C (PA < 140/90 mmHg ou PA diurna < 135/85 mmHg) e 130 com HAR-NC (PA ≥ 140 ou 90 mmHg e PA diurna > 135 ou 85 mmHg). Durante o seguimento (4,98 ± 2,9 anos), as taxas de eventos por 100 pacientes/ano foram de 0,87 e 4,1, respectivamente. Esses dados mostraram também que pacientes com HAR-NC têm maior risco de DAC, acidente vascular encefálico, arteriopatia, insuficiência cardíaca congestiva (ICC), doença renal e mortes de todas as causas quando comparados a pacientes com HAR-C.

6.4. Fenótipo do Paciente com Hipertensão Arterial Refratária

A hipertensão arterial refratária parece ser um fenótipo extremo do hipertenso resistente. Recentemente, a caracterização fenotípica mostrou que esses pacientes são mais jovens do que os resistentes em geral, mais comumente mulheres, com maior frequência de insuficiência cardíaca e, de maneira destacada, têm maior atividade simpática do que os pacientes resistentes.^5^ Esses achados são importantes pilares para a fisiopatologia da refratariedade, constituindo potencialmente um alvo terapêutico para procedimentos como a desnervação renal. Estudos nesta área estão atualmente em desenvolvimento.

## 7. Causas Secundárias de Hipertensão Arterial Resistente

**Coordenadora:** Fernanda Marciano Consolim-Colombo

**Autores:** Márcio Gonçalves de Sousa, Flávio Antonio de Oliveira Borelli, Cibele Isaac Saad Rodrigues e Fernanda Marciano Consolim-Colombo

### 7.1. Introdução

A hipertensão arterial secundária (HASec) é definida como um aumento da PA devido a uma causa identificável.^33 , 105^ Frente a um paciente com HAR, torna-se imperativa a investigação das causas mais prevalentes de HASec “não endócrina” e “endócrina” após exclusão de uso de fármacos que podem interferir com os valores pressóricos: anti-inflamatórios, glicocorticoides, descongestionantes nasais, inibidores de apetite, antidepressivos, imunossupressores, eritropoietina, contraceptivos e drogas ilícitas.^33 , 105^

### 7.2. Hipertensão Arterial Secundária de Causas Não Endócrinas

#### 7.2.1. Apneia Obstrutiva do Sono

Definida como cessação total ou parcial do fluxo respiratório durante o sono, esta síndrome promove dessaturação da oxi-hemoglobina e microdespertares durante o sono. Estima-se que a prevalência de AOS seja de 17% dos adultos americanos^106^ e 30% da população de hipertensos, podendo afetar 60 a 80% dos hipertensos resistentes.^94^ Uma recente metanálise^107^concluiu que a presença de AOS está relacionada a maior risco de HAR.^107^

A ativação do sistema nervoso simpático e as alterações humorais são responsáveis por modificações na integridade do endotélio vascular, e suas consequências nos pacientes com AOS incluem aumento da PA, desenvolvimento de doença aterosclerótica, arritmias cardíacas, dentre outras.^108^ A suspeita clínica pode ser rastreada a partir do questionário de Berlin.^109^

O diagnóstico é feito com o exame de polissonografia, que registra os índices de apneia/hipopneia superiores a cinco eventos/hora.

No tratamento, devem ser instituídas orientações sobre higiene do sono e perda de peso, dentre outras; para a desobstrução das vias de respiração, o uso de equipamento que produz pressão positiva contínua nas vias respiratórias (CPAP, *continuous positive airway pressure* ) é o mais difundido. No entanto, o impacto desse tratamento na redução dos valores de PA ainda é matéria de debate.^110 , 111^

#### 7.2.2. Doença do Parênquima Renal

A doença do parênquima renal (DPR) é uma das causas mais prevalentes de HASec. Seu diagnóstico é relativamente simples, pois a investigação da função dos rins faz parte da abordagem de rotina do hipertenso. A hipertensão arterial tem alta prevalência nos pacientes em diálise e nos transplantados renais, e os eventos CV são os responsáveis pela alta morbimortalidade dessa população.^112^

A progressão da disfunção renal nos portadores de DPR é diretamente relacionada aos valores pressóricos, e as metas pressóricas devem ser alcançadas para reduzir a morbimortalidade CV. Nos portadores de DPR e nos transplantados renais, os IECA e os antagonistas dos receptores da angiotensina II demonstraram proteção adicional aos rins, além da obtida pela redução do nível pressórico, sendo, por isso, os fármacos preferenciais.^33 , 105 , 113^

#### 7.2.3. Estenose da Artéria Renal

A doença renovascular é o termo usado para definir o acometimento das artérias renais por diferentes patologias, como doença aterosclerótica, displasia fibromuscular e vasculites, que podem levar à obstrução dos vasos. Quando há discreta obstrução arterial, comumente não há sintomas associados; porém, quando há obstruções superiores a 70% da artéria, pode ocorrer hipertensão arterial grave e até mesmo nefropatia isquêmica.

A estenose da artéria renal (EAR) de origem aterosclerótica está presente em 12,5% dos pacientes hipertensos resistentes com idade acima de 50 anos.^114^ O diagnóstico deve sempre ser feito, mas o manuseio dessa condição ainda é muito discutido na literatura.^115 , 116^ O controle pressórico adequado e o bloqueio na progressiva deterioração da função renal são os objetivos principais do tratamento desses pacientes. Para isso, duas são as possibilidades terapêuticas nesta população: clínica ou intervencionista (cirúrgica ou percutânea, com ou sem implante de próteses vasculares [ *stents* ]).

As intervenções são recomendadas para os pacientes com HAR ou hipertensão arterial acelerada com perda progressiva da função renal, com estenose bilateral ou com estenose em rim “único”, ou com graves complicações (ICC e edema agudo de pulmão de repetição).^33 , 115 , 116^

Outras potenciais indicações cirúrgicas são: obstrução total da artéria renal, grandes fístulas arteriovenosas, lesão de aorta englobando as artérias renais e insucesso no tratamento clínico ou endovascular.^117^

## 7.3. Hipertensão Arterial Secundária de Causas Endócrinas

### 7.3.1. Hiperaldosteronismo Primário

Considerado no passado como um tipo raro de HASec (prevalência na ordem de 1%), atualmente, julga-se que o hiperaldosteronismo pode chegar até 22% em populações com HAR.^118 , 119^ O adenoma da suprarrenal é a causa mais frequente, sendo a hiperplasia uni ou bilateral menos detectadas. Carcinomas, apesar de infrequentes, ou formas genéticas, também podem ser responsáveis pela instalação da doença.

A aldosterona, por meio da ativação dos receptores mineralocorticoides, está relacionada à resistência insulínica e à disfunção endotelial; consequentemente, ela participa do desenvolvimento da síndrome metabólica e das lesões CV e renais associadas ao quadro de HAR. Assim, o bloqueio desses receptores mineralocorticoides promove melhora da disfunção endotelial e contribui para melhor resposta ao tratamento da HAR e das LOA.^118 , 119^

Na realização do diagnóstico, todos os portadores de HAR (não apenas aqueles que apresentem hipocalemia) devem ser avaliados quanto à ocorrência de hiperaldosteronismo.^33^ A triagem inclui avaliação da razão aldosterona plasmática (expressa em ng/dL) pela atividade de renina plasmática (expressa ng/mL/h) (AP/ARP). Esse método tem grande sensibilidade, mas pode apresentar resultados falso-positivos. Desse modo, recomenda-se utilizar como valores mínimos de AP e de ARP, respectivamente, 15 ng/dL e 0,5 ng/mL/h. Se a razão AP/ARP for ≥ 100, o diagnóstico será de hiperaldosteronismo; valores < 20 a 30 indicam baixa probabilidade; e valores entre esses extremos detectam “potenciais portadores” dessa condição.^120^Nesse último caso, testes para avaliação do eixo renina-aldosterona (prova de infusão de volume, caminhada, uso de diuréticos) podem ser realizados.

Para identificação de adenomas ou hiperplasia na suprarrenal por imagem, usa-se a tomografia ou a ressonância magnética. A ausência de um tumor visível à tomografia não exclui um microadenoma, daí a importância da procura de um excesso na produção de aldosterona. Imagens funcionais, obtidas pela cintilografia de adrenal, podem ser úteis na detecção dos adenomas, podendo diferenciá-los das hiperplasias nodulares em até 90% dos casos. A coleta de amostra de sangue na veia suprarrenal pode ser utilizada para confirmar a lateralização na secreção de aldosterona e a presença de adenoma unilateral.^120 , 121^

Quanto ao tratamento, na presença de adenoma unilateral, a ressecção unilateral geralmente corrige a produção excessiva de aldosterona e a perda de potássio. A resposta da PA ao tratamento cirúrgico é variável. As hiperplasias são beneficiadas com o bloqueio dos receptores de aldosterona.^121^

### 7.3.2. Feocromocitoma

O feocromocitoma é um tumor neuroendócrino raro, originário de células cromafins (produtoras de catecolaminas), cuja manifestação clínica mais comum é a elevação da PA, podendo ser originário da medula adrenal ou de paragânglios extra-adrenais (paragangliomas). Seu pico de exacerbação clínica está entre a terceira e quarta décadas de vida, mas 10% dos casos surgem na infância.

O tumor pode apresentar-se de modo esporádico ou associado a síndromes genéticas.^122 , 123^ Geralmente é unilateral; porém, nas síndromes familiares, pode ser bilateral, múltiplo e extra-adrenal, benigno ou maligno (5 a 26% dos casos). Essa etiologia deve ser investigada em todos os pacientes que apresentem HAR e/ou sintomas ou sinais sugestivos de liberação adrenérgica. A hipertensão paroxística ocorre em 30% dos casos, sendo desencadeada por atividades físicas habituais, exercícios mais intensos, procedimentos cirúrgicos e pelo uso de algumas substâncias, como antidepressivos tricíclicos, histamina e opiáceos. Os paroxismos podem ser acompanhados de cefaleia (60 a 90%), sudorese (55 a 75%) e palpitações (50 a 70%). Sintomas de insuficiência cardíaca e alterações no eletrocardiograma podem ser indicativos de miocardite induzida por excesso de catecolaminas.

No diagnóstico, a dosagem de metanefrinas (metabólitos das catecolaminas), tanto no plasma quanto na urina de 24 h, apresenta maior sensibilidade e especificidade que a dosagem direta de catecolaminas. Quando os exames laboratoriais não forem elucidativos, o teste de supressão com clonidina pode ser realizado (administração de 0,2 mg de clonidina com dosagem de catecolaminas 1 h antes e 2 h após a ingestão do fármaco).

Para o diagnóstico topográfico dos tumores e eventualmente de metástases, os métodos de imagens recomendados são tomografia computadorizada e ressonância magnética, ambas com sensibilidade próxima a 100% para tumores adrenais. O mapeamento de corpo inteiro com metaiodobenzilguanidina (MIBG) 131 ou 121 apresenta sensibilidade de 56 a 85% (tumores malignos) e alta especificidade. O octreoscan, o mapeamento ósseo e o PET scan (com diferentes marcadores) podem ser decisivos quando os exames de localização anteriores são negativos ou na investigação de doença maligna.

O tratamento é cirúrgico. Porém, na terapia medicamentosa pré-operatória ou crônica, são usados inicialmente alfabloqueadores (prazosin, doxazocin e dibenzilina), combinados ou não a outros agentes, como betabloqueadores (após alfabloqueio efetivo), IECA e BCC. Para a intervenção cirúrgica, recomenda-se controle prévio dos níveis de PA e reposição volêmica.^124^ Em crises agudas e durante a cirurgia, nitroprussiato de sódio pode ser utilizado.^124^

### 7.3.3. Hipotireoidismo e Hipertireoidismo

A hipertensão arterial pode estar presente em 40% dos portadores de distúrbios da tireoide, pois a correção da disfunção glandular geralmente é responsável pelo controle da PA.^125^ Uma vez corrigido o hipo ou o hipertireoidismo, e persistindo níveis elevados de PA, está indicado o uso de fármacos anti-hipertensivos.^32 , 126^

As causas de HASec em pacientes com HAR são sumarizadas na Tabela 1 .

**Tabela 1:** – Prevalência, achados clínicos e estudos adicionais das causas mais comuns de hipertensão arterial secundária em pacientes com hipertensão arterial resistente

Causa secundária	Prevalência geral	Prevalência na HAR	Achados clínicos	Investigação diagnóstica
Apneia obstrutiva do sono^94,107,109^	> 5 a 15%	> 30%	Ronco, sonolência diurna, cefaleia matinal, síndrome metabólica	Questionário de Berlim, *stop-bang* , Escala de sonolência de Epworth Polissonografia (padrão-ouro) ou polissonografia residencial. Diagnóstico. Índice apneia e/ou hipopneia > 5 eventos por hora de sono
Doença do parênquima renal^113^	1,6 a 8%	2 a 10%	Edema, anorexia, nictúria, fadiga, anemia, ureia e creatinina elevadas, alterações do sedimento urinário	Exame de urina (densidade baixa, hematúria glomerular ou albuminúria), cálculo do RFG estimado, US renal, Pesquisa de albuminúria e relação proteinúria/creatininúria em amostra isolada
Estenose da artéria renal^115,116^	1 a 8%	2,5 a 20%	Sopro abdominal, edema agudo de pulmão, alteração da função renal por medicamentos que bloqueiam o SRAA, rins assimétricos	Rastreio: US com Doppler de artérias renais (operador dependente) e/ou renograma com ou sem captopril, angiorressonância, tomografia computadorizada, arteriografia renal convencional (padrão-ouro)
Hiperaldosteronismo primário^119-121^	1,4 a 10%	6 a 23%	Maioria assintomática HAR hipopotassemia (não obrigatória e não habitual) Nódulo adrenal incidental	Relação AP/ARP > 30 na ausência de antagonistas de aldosterona. Testes confirmatórios (supressão com fludrocortisona ou infusão salina) Exames de imagem: tomografia computadorizada helicoidal com cortes finos (preferencial) ou ressonância magnética
Doenças da tireoide^32^ Hipotiroidismo	1 a 2%	1 a 3%	Fadiga, ganho de peso, perda de cabelo, hipertensão arterial sistólica, fraqueza muscular.	TSH e T4 Livre
Hipertiroidismo			Intolerância ao calor, perda de peso, hipertensão arterial diastólica, palpitações, exoftalmia, tremores, taquicardia	
Síndrome de Cushing^32^	0,5%	< 1%	Ganho de peso, fadiga, hirsutismo, amenorreia, “fácies em lua cheia”, “corcova dorsal”, estrias purpúricas, obesidade central, hipopotassemia	Cortisol salivar Cortisol urinário de 24 horas Cortisol matinal (8 horas) e 8 horas após administração de dexametasona (1 mg) às 24 h. Ressonância magnética
Feocromocitoma^127,128^	0,2 a 0,5%	< 1%	Hipertensão arterial episódica, lábil ou resistente, paroxismos de cefaleia, sudorese profusa e palpitações, palidez	Metanefrinas plasmáticas livres e/ou urinárias de 24 h (valores o dobro ou triplo do normal), catecolaminas plasmáticas e/ou urinárias de 24 h e/ou tomografia computadorizada e ressonância magnética
Coarctação de aorta^129^	< 1%	< 1%	Diferença de PAS/PAD > 20/10 mmHg entre membros superiores e inferiores; sopro ejetivo em região interescapular	Entalhe da borda inferior da costela na radiografia de tórax, rastreio com ecodopplercardiograma, ressonância magnética ou angiografia da aorta torácica

*Adaptada de Rimoldi SF et al.^105^ AP/ARP: aldosterona plasmática/atividade de renina plasmática; HAR: hipertensão arterial resistente; PAD: pressão arterial diastólica; PAS: pressão arterial sistólica; RFG: ritmo de filtração glomerular; SRAA: sistema renina-angiotensina-aldosterona; US: ultrassonografia.*

## 8. Tratamento Não Farmacológico

**Coordenador:** Sérgio Emanuel Kaiser

**Autores:** Gil Fernando Salles, Maria de Fátima de Azevedo e Lucélia Batista Neves Cunha Magalhães.

### 8.1. Perda Ponderal

Vários mecanismos favorecem a manutenção de uma PA elevada em hipertensos obesos, como AOS, hiperatividade simpática, disfunção endotelial, modificação da microbiota intestinal, todos capazes de promover um fenótipo inflamatório e perpetuar um ciclo vicioso.^130^ Pacientes com índice de massa corporal (IMC) ≥ 30 kg/m^2^ têm 50% mais chances de apresentar PA não controlada do que aqueles com IMC normal (< 25 kg/m^2^).^131^ Um IMC > 40 kg/m^2^ triplica as chances de se requererem múltiplos fármacos para controle da PA.^132^

Uma perda ponderal de 10 kg associa-se a uma redução média de 6,0 mmHg na PA sistólica e 4,0 mmHg na PA diastólica.^133^ Surpreendentemente, não há evidências consistentes sobre o efeito da perda de peso induzida por dieta em hipertensos resistentes, mas essa recomendação atende ao bom senso e às evidências disponíveis nos demais subgrupos. Também não existem dados sobre o efeito da cirurgia bariátrica sobre a PA nesse subgrupo. Recente estudo aleatorizado demonstrou redução de ao menos 30% no número de anti-hipertensivos em 84% dos pacientes operados, em comparação a 12,4% do grupo tratado clinicamente.^134^

### 8.2. Restrição de Sal

O controle no consumo de sal é especialmente eficaz em idosos, afrodescendentes e indivíduos com filtração glomerular diminuída.^135^ Nessas situações, restringe-se a capacidade de excreção de água e sódio pelos rins, tornando a PA mais dependente de variações volêmicas. Não por acaso, a sensibilidade ao sódio e a sobrecarga volêmica respondem pelo principal mecanismo fisiopatológico da maioria dos casos de HAR.^136^ Uma revisão sistemática e metanálise envolvendo 34 estudos com 3.230 participantes, sobre o efeito da redução em longo prazo na ingestão de sódio, revelou queda na PA sistólica de 5,8 mmHg (2,5 a 9,2; p = 0,001) associada a redução da excreção urinária de sódio até 100 mmol em 24 h, o que corresponde a uma diminuição da ingestão de sal de aproximadamente 6 g/dia.^137^ Em hipertensos resistentes, uma dieta hipossódica com 2,5 g diários de sal foi capaz de reduzir a PA em até 23,0/9,0 mmHg, em clara demonstração da eficácia dessa medida, não obstante a possibilidade de comprometimento da aderência a longo prazo a tão acentuada restrição no consumo de sal.^79^

### 8.3. Ingestão de Álcool

Fruto da relação direta entre a quantidade de álcool consumida e os níveis pressóricos, o consumo excessivo de álcool contribui significativamente para a dificuldade no controle da PA;^138^ afinal, o consumo diário de mais de dois “ *drinks* ” (cerca de 24 g/dia) associa-se à elevação dos níveis pressóricos.^139^ Recente metanálise de 36 estudos com 2.865 participantes revelou que a redução de 50% na ingestão diária de álcool entre os consumidores de seis ou mais “ *drinks* ” (72 g) promoveu queda de 5,50 mmHg na PA sistólica (IC 95%; 6,70 a 4,30) e de 3,97 mmHg na PA diastólica (IC 95%; 4,70 a 3,25).^140^ Não há estudos publicados em hipertensos resistentes; porém, com base nas informações disponíveis, recomenda-se a restrição do consumo diário de álcool a menos de dois “ *drinks* -padrão” (cerca de 24 g) ou até sua cessação.

### 8.4. Atividade Física

Apesar de avaliada apenas em pequenos grupos de hipertensos resistentes, a atividade física é provavelmente tão ou mais benéfica nestes do que em não resistentes.^40 , 141^Exercício aeróbico regular diminui a PA de consultório e a ambulatorial em hipertensos resistentes,^142 - 145^ além de atenuar a característica ativação neuro-humoral.^146^ Não obstante a inexistência de estudos sobre exercício resistido nesse subgrupo, supõe-se haver vantagem ao menos semelhante à observada em hipertensos não resistentes.^147^ Além disso, a melhor capacidade cardiorrespiratória obtida com atividade física parece reduzir a mortalidade de hipertensos resistentes.^148^ Portanto, essa categoria de pacientes deve ser incentivada a realizar atividade física regular de moderada intensidade sob supervisão adequada. Naqueles com PA muito elevada (PA sistólica ≥ 180 mmHg ou PA diastólica ≥ 110 mmHg), a atividade física deve ser adiada até que a otimização do tratamento medicamentoso promova a redução da PA.^40 , 141^

## 9. Tratamento Farmacológico da Hipertensão Arterial Resistente

**Coordenador:** Rui Manoel dos Santos Póvoa

**Autores:** Marcus Vinícius Bolívar Malachias, Armando da Rocha Nogueira e Paulo César Brandão Veiga Jardim

O objetivo do tratamento medicamentoso na HAR é detectar as causas do não controle e encontrar a melhor combinação de fármacos, visando o alcance das metas pressóricas com menor ocorrência de efeitos adversos e maior adesão.

Em geral, busca-se otimizar o tratamento tríplice com os fármacos preferenciais, que são: IECA ou BRA, BCC di-hidropiridínico e DT.^33 , 149^

Os IECA ou BRA, por serem mais bem tolerados, precisam ser elevados às doses máximas na HAR. Deve ser utilizado um DT de longa ação e maior potência, como a clortalidona em lugar da hidroclorotiazida, em doses adequadas ao controle da volemia, de 12,5 a 50 mg, em dose única pela manhã.^1 , 33 , 40 , 150^ A indapamida constitui uma segunda opção de DT na HAR.^150^ A furosemida deve ser utilizada em casos de DRC, com RFG igual ou inferior a 30 ml/min.^1 , 33^ Na HAR, o BCC deve ser preferencialmente, tomado à noite, para que haja alternância de picos de ação dos anti-hipertensivos.^40^

A intolerância aos BCC, devido a efeitos colaterais, muitas vezes é uma das causas de resistência ao tratamento. Nesses casos, pode ser tentada a utilização de BCCs lipofílicos (manidipino, lercanidipino, manidipino) ou o levanlodipino, em baixas doses, ou, em casos selecionados, um BCC não di-hidropiridínico, como diltiazem e verapamil.^33^ Na impossibilidade de uso de um BCC, pode ser considerada a introdução de um betabloqueador, preferencialmente com ação vasodilatadora, como nebivolol ou carvedilol.^33 , 151^ Betabloqueadores também podem ser considerados em associação a um ou mais anti-hipertensivos preferenciais – IECA ou BRA, DT, BCC – em condições especiais como insuficiência cardíaca, coronariopatia, frequência cardíaca basal elevada, entre outras.^33 , 150 , 151^

O não alcance da meta pressórica com o esquema tríplice exige a utilização de um 4^o^ fármaco, cuja opção preferencial atual é a espironolactona, de 25 a 50 mg ao dia.^13 , 152 - 154^ Em casos de intolerância à espironolactona, cujo efeito adverso principal é a ocorrência de ginecomastia em homens, pode ser tentada a utilização de 12,5 mg ao dia. Como não há disponibilidade de eplerenone em nosso meio, caso persista a intolerância à espironolactona, mesmo em baixas doses, deverá ser avaliada a sua substituição por um simpatolítico central, preferencialmente a clonidina, de 0,100 a 0,200 mg, duas vezes ao dia,^152^ ou um diurético poupador de potássio, preferencialmente a amilorida (só disponível em nosso meio de forma isolada em formulações magistrais), de 10 a 20 mg;^155^ ou um betabloqueador, preferencialmente com ação vasodilatadora, se não tiver sido ainda empregado;^40^ ou um alfa bloqueador, preferencialmente a doxazosina, em dosagem de 1 a 16 mg, em uma tomada (noturna) ou duas tomadas diárias.^33 , 40 , 155^

**Figura 4:**
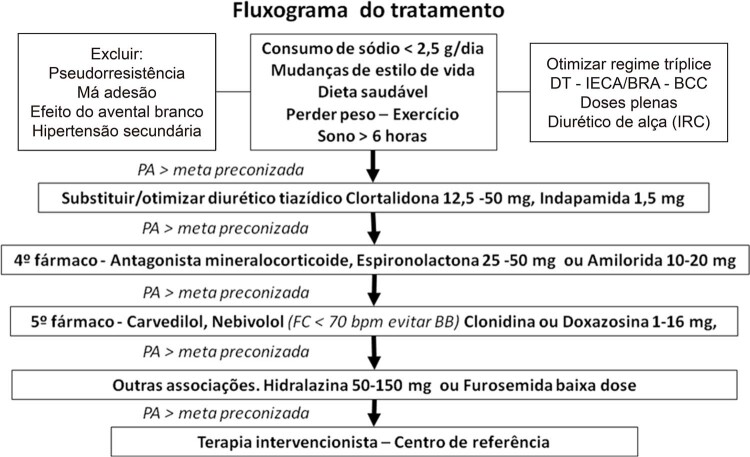
– *Fluxograma de tratamento de HA. BCC: bloqueador dos canais de cálcio; BRA: bloqueador do receptor de angiotensina; FC: frequência cardíaca; IECA: inibidor da enzima conversora da angiotensina; IRC: insuficiência renal crônica; PA: pressão arterial.*

Todos esses anti-hipertensivos podem ser utilizados em associações, quando necessário, para o controle pressórico.^33^ Caso não se obtenha o controle com a adição do 4º fármaco ou com combinações das opções subsequentes, deve-se utilizar um vasodilatador direto, preferencialmente a hidralazina, em doses diárias de 50 a 150 mg, fracionadas em 2 a 3 tomadas.^40^ O vasodilatador minoxidil, em face de seus frequentes efeitos adversos, deve ser reservado para situações muito resistentes, quando há falha de todas as alternativas anteriores^40 , 150^ ( Figura 4 ).

No tratamento da HAR, deve-se estar atento aos possíveis efeitos adversos de cada um dos fármacos empregados, assim como às suas possíveis interações medicamentosas.

## 10. Novos Tratamentos da Hipertensão Arterial Resistente

**Coordenador:** Luiz Aparecido Bortolotto

**Autores:** Luiz Aparecido Bortolotto, Luciano Ferreira Drager e Thiago de Souza Veiga Jardim

### 10.1. Introdução

Nos últimos anos, novas formas de tratamento intervencionista têm sido avaliadas para pacientes com HAR, tais como:

### 10.2. Estimulação Direta do Seio Carotídeo

O estímulo dos barorreceptores carotídeos leva ao aumento da atividade dos mesmos e, consequente, redução do fluxo simpático, resultando em diminuição da PA.^156^ Intervenções que promovem esta estimulação têm sido usadas para o tratamento de pacientes com HAR não responsivos a tratamento clínico.^156 - 159^ A terapia de ativação do barorreflexo (TAB) é um procedimento cirúrgico onde eletrodos são implantados cirurgicamente na porção externa do seio carotídeo bilateral ou unilateral.^157 , 159^ A TAB mostrou reduções significativas da PA, que persistem por até 3 anos em estudos randomizados e controlado.^157 , 159^ Entretanto, o procedimento é invasivo, de alto custo e apresenta efeitos colaterais, que restringem sua indicação na prática clínica.^156 , 159^ Outra forma de estímulo é a amplificação do barorreflexo endovascular (implante de dispositivo expansível dentro da carótida), que demonstrou resultados promissores no controle da PA na HAR, com maior segurança.^156^ Estes procedimentos não estão disponíveis no Brasil.

### 10.3. Denervação Simpática Renal

A denervação simpática renal (DSR) por cateter de ablação reduz a atividade eferente renal, com consequente aumento do fluxo sanguíneo renal, diminuição da ativação do sistema renina-angiotensina-aldosterona e da retenção de água, e também da atividade aferente renal que, através de sinais cerebrais, diminui ação simpática sobre coração e vasos.^160^

Dados obtidos em estudos não controlados mostraram reduções de até 30 mmHg na PA sistólica de consultório em pacientes com HAR, sem complicações do procedimento.^161^ Entretanto, o estudo SYMPLICITY HTN-3,^162^ randomizado e controlado com procedimento *sham* , não mostrou efeito significativamente superior de redução da PA após 6 meses com a DSR. Uma metanálise com 11 estudos controlados comparando DSR com tratamento medicamentoso otimizado ou procedimento *sham* em pacientes com HAR não demonstrou superioridade da DSR em reduzir a PA, havendo heterogeneidade de respostas nos estudos devido principalmente a falta de controle sham na maioria das publicações e heterogeneidade na avaliação da adesão ao tratamento.^163^

O desenvolvimento de novos cateteres circunferenciais com aplicações distais nas artérias renais pode promover DSR mais completa, e efeitos de redução da PA têm sido demonstrados em pacientes hipertensos não tratados.^164^

No posicionamento de 2018 da Sociedade Europeia de Hipertensão, DSR não é recomendada em geral para o tratamento de hipertensão arterial, mas há recomendações para a sua realização no contexto de estudos clínicos controlados com procedimento *sham* e terapia otimizada para avaliação da segurança e da eficácia em população com grande número de indivíduos.^160^

Com base nessas evidências, no momento, a DSR seria uma alternativa apenas para pacientes com HAR-NC com tratamento farmacológico otimizado e comprovada adesão terapêutica ou com importantes efeitos adversos das medicações, sempre em centros de referência treinados para o procedimento.^164^

### 10.4. Uso de Pressão Positiva Contínua em Vias Aéreas

A AOS é uma condição clínica presente em mais da metade dos pacientes com HAR^94^ e seu principal tratamento é o CPAP, um compressor de ar que provoca pressão positiva contínua na via aérea do paciente. Até o momento, sete estudos randomizados analisaram o efeito do tratamento da AOS com CPAP em pacientes com HAR.^165 - 171^ Com exceção de um deles,^170^ os demais encontraram reduções significativas de PA (5 mmHg em média; um dos estudos mostrou reduções ≥ 10 mmHg após uso do CPAP).^169^

No entanto, a proporção de pacientes que alcançou a meta de PA (< 140/90 mmHg) com o CPAP é baixa, possivelmente explicada pela pouca adesão ao CPAP. Na prática clínica observa-se que a resposta da PA ao CPAP pode ser variável, mesmo em pacientes com boa adesão. Recente estudo mostrou biomarcadores preditores de melhor resposta da PA ao uso do CPAP em pacientes com HAR.^172^ A validação e a aplicação em larga escala desses biomarcadores podem ajudar a selecionar melhor os pacientes que terão mais benefícios com a redução da PA.

#### 10.5. Fístula Arteriovenosa

A aplicação de uma fístula arteriovenosa (FAV) pode promover diminuição da PA por mecanismos relacionados a: redução na resistência periférica total e no volume sanguíneo, inibição de barorreflexo e liberação de peptídeos natriuréticos.^173^ Em estudo prospectivo randomizado e controlado, a criação de FAV ilíaca–central por um dispositivo implantável em 44 pacientes com HAR foi acompanhada de significativa redução da PA sistólica de consultório e ambulatorial de 24 h, quando comparada a tratamento medicamentoso.^174^ Entretanto, houve uma taxa elevada de complicações devido a estenose venosa ipsilateral, necessitando de intervenção no grupo com a FAV.

Novos estudos com maior número de pacientes e comparações da FAV com o procedimento *sham* estão sendo realizados para comprovar os benefícios dela na HAR.^173^
