# Supplementary material for: Posicionamento Brasileiro sobre Hipertensão Arterial Resistente – 2020
Source: Arq Bras Cardiol. 2020 Apr 6;114(3):576–96. [Article in Portuguese] doi: 10.36660/abc.20200198 (PMC7792719; doi:10.36660/abc.20200198)
Supplement: Translation

# Translation: Brazilian Position Statement on Resistant Hypertension – 2020

**Authors:** Juan Carlos Yugar-Toledo, Heitor Moreno, Miguel Gus, Guido Bernardo Aranha Rosito, Luiz César Nazário Scala, Elizabeth Silaid Muxfeldt, Alexandre Alessi, Andrea Araújo Brandão, Osni Moreira, Audes Diógenes de Magalhães Feitosa, Oswaldo Passarelli, Dilma do Socorro Moraes de Souza, Celso Amodeo, Weimar Kunz Sebba Barroso, Marco Antônio Mota Gomes, Annelise Machado Gomes de Paiva, Eduardo Costa Duarte Barbosa, Roberto Dischinger Miranda, José Fernando Vilela-Martin, Wilson Nadruz, Cibele Isaac Saad Rodrigues, Luciano Ferreira Drager, Luiz Aparecido Bortolotto, Fernanda Marciano Consolim-Colombo, Márcio Gonçalves de Sousa, Flávio Antonio de Oliveira Borelli, Sérgio Emanuel Kaiser, Gil Fernando Salles, Maria de Fátima de Azevedo, Lucélia Batista Neves Cunha Magalhães, Rui Manoel dos Santos Póvoa, Marcus Vinícius Bolívar Malachias, Armando da Rocha Nogueira, Paulo César Brandão Veiga Jardim, Thiago de Souza Veiga Jardim

**Affiliations:** 1 Brazil Faculdade Estadual de Medicina de São José do Rio Preto, São José do Rio Preto, SP – Brazil; 2 Brazil Faculdade de Ciências Médicas da Universidade Estadual de Campinas, Campinas, SP – Brazil; 3 Brazil Hospital Moinhos de Vento, Porto Alegre, RS – Brazil; 4 Brazil Universidade Federal de Ciências da Saúde de Porto Alegre, Porto Alegre, RS – Brazil; 5 Brazil Faculdade de Medicina da Universidade Federal de Mato Grosso, Cuiabá, MT – Brazil; 6 Brazil Hospital Universitário Clementino Fraga Filho, Universidade Federal do Rio de Janeiro, Rio de Janeiro, RJ – Brazil; 7 Brazil Universidade Federal do Paraná, Curitiba, PR – Brazil; 8 Brazil Universidade do Estado do Rio de Janeiro, Rio de Janeiro, RJ – Brazil; 9 Brazil Pontifícia Universidade Católica do Paraná, Curitiba, PR – Brazil; 10 Brazil Universidade Federal de Pernambuco, Recife, PE – Brazil; 11 Brazil Instituto Dante Pazzanese de Cardiologia, São Paulo, SP – Brazil; 12 Brazil Faculdade de Medicina da Universidade Federal do Pará, Belém, PA – Brazil; 13 Brazil Faculdade de Medicina da Universidade Federal de Goiás, Goiânia, GO – Brazil; 14 Brazil Centro Universitário CESMAC, Maceió, AL – Brazil; 15 Brazil Liga de Combate à Hipertensão de Porto Alegre, Porto Alegre, RS – Brazil; 16 Brazil Universidade Federal de São Paulo, São Paulo, SP – Brazil; 17 Brazil Faculdade de Ciências Médicas e da Saúde Pontifícia Universidade Católica de são Paulo, São Paulo, SP – Brazil; 18 Brazil Instituto do Coração do Hospital das Clínicas da Faculdade Medicina Universidade de São Paulo, São Paulo, SP – Brazil; 19 Brazil Universidade Federal do Rio Grande do Norte, Natal, RN – Brazil; 20 Brazil Faculdade de Tecnologia e Ciências da Universidade Federal da Bahia, Salvador, BA – Brazil; 21 Brazil Faculdade de Ciências Médicas de Minas Gerais, Belo horizonte, MG – Brazil; 22 Brazil Universidade Federal do Rio de Janeiro, Rio de Janeiro, RJ – Brazil; 23 Brazil Faculdade de Medicina da Universidade Federal de Goiás, Goiânia, GO – Brazil; 24 Brazil Hospital do Coração de Goiás, Goiânia, GO – Brazil

Development: Department of Hypertension (DH) of the Brazilian Society of Cardiology

Norms and Guidelines Council (2020-2021): Brivaldo Markman Filho, Antonio Carlos Sobral Sousa, Aurora Felice Castro Issa, Bruno Ramos Nascimento, Harry Correa Filho, Marcelo Luiz Campos Vieira

Norms and Guidelines Coordinator (2020-2021): Brivaldo Markman Filho

**Table:** 

Declaration of potential conflict of interests of authors/collaborators of the Brazilian Position Statement on Resistant Hypertension – 2020 If, within the last 3 years, the author/collaborator of the statement:							
Names of statement collaborators	Participated in clinical and/or experimental studies sponsored by pharmaceutical or equipment companies related to this guideline	Spoke at events or activities sponsored by industry related to this guideline	Was (is) a member of a board of advisors or a board of directors of a pharmaceutical or equipment industry	Participated in normative committees of scientific research sponsored by industry	Received personal or institutional funding from industry	Wrote scientific papers in journals sponsored by industry	Owns stocks in industry
Alexandre Alessi	No	No	No	No	No	No	No
Andrea Araújo Brandão	No	Abbott, EMS, Merck	No	No	Servier, Medley	Servier, Abbott	No
Annelise Machado Gomes de Paiva	No	No	No	No	No	No	No
Armando da Rocha Nogueira	No	No	No	No	No	No	No
Audes Diógenes de Magalhães Feitosa	No	Omron	Omron	No	No	No	No
Celso Amodeo	Medtronic	No	No	No	Novo Nordisk	No	No
Cibele Isaac Saad Rodrigues	No	No	No	No	No	No	No
Dilma do Socorro Moraes de Souza	No	No	No	No	No	No	No
Eduardo Costa Duarte Barbosa	No	No	No	No	EMS, Servier	EMS, Novartis, Medley	No
Elizabeth Silaid Muxfeldt	No	No	No	No	No	No	No
Fernanda Marciano Consolim-Colombo	No	Servier, Merck	No	No	Servier, Merck, Daiichi Sankyo	Servier, Merck	No
Flavio Antonio de Oliveira Borelli	No	No	No	No	No	Libbs	No
Gil Fernando Salles	No	No	No	No	No	No	No
Guido Bernardo Aranha Rosito	No	No	No	No	No	No	No
Heitor Moreno Júnior	No	No	No	No	No	No	No
José Fernando Vilela-Martin	No	No	No	No	No	No	No
Juan Carlos Yugar-Toledo	No	No	No	No	No	No	No
Luciano Ferreira Drager	No	No	No	No	No	No	No
Lucélia Batista Neves Cunha Magalhães	No	No	No	No	No	No	No
Luiz Aparecido Bortolotto	No	No	No	No	No	Servier, Merck	No
Luiz César Nazário Scala	No	No	No	No	No	No	No
Márcio Gonçalves de Sousa	No	No	No	No	No	No	No
Marco Antônio Mota Gomes	No	No	Servier, Torrent, Abbott, Omron, Novartis, Astrazeneca	Não	Servier, Torrent, Abbott, Omron, Novartis, Astrazeneca	Servier, Torrent, Abbott, Omron, Novartis, Astrazeneca	No
Marcus Vinícius Bolívar Malachias	No	Abbott, Biolab, Libbs, Novo Nordisk, Takeda	No	No	No	Abbott, Biolab, Farmoquímica, Libbs, Novo Nordisk	No
Maria de Fátima de Azevedo	No	No	No	No	No	No	No
Miguel Gus	No	No	No	No	No	No	No
Osni Moreira Filho	No	Servier, Merck	No	No	No	No	No
Oswaldo Passarelli Júnior	No	No	No	No	No	No	No
Paulo César Brandão Veiga Jardim	No	No	No	No	No	Biolab, Aché, Libbs	No
Roberto Dischinger Miranda	No	No	No	No	No	No	No
Rui Manoel dos Santos Póvoa	No	No	No	No	No	No	No
Sérgio Emanuel Kaiser	No	No	No	No	No	Novartis	No
Thiago de Souza Veiga Jardim	No	Torrent	No	No	Libbs, Torrent, Novartis	Torrent	No
Weimar Kunz Sebba Barroso	Amgen, AstraZeneca, Torrent, EMS, Novartis	No	No	No	EMS, Sandoz, Servier, Novartis	Medley, Sandoz, EMS	No
Wilson Nadruz Júnior	No	No	No	No	No	No	No

Content

1. Definition and Epidemiology 580

1.1. Definition/New Concepts 580

1.2. Control of Hypertension in Brazil and Worldwide 580

1.3. Incidence and Prevalence of Resistant Hypertension 580

1.4. Factors Related to Resistant Hypertension 580

2. Prognostic Aspects 581

2.1. Introduction 581

2.2. Office Blood Pressure and Ambulatory Blood Pressure Monitoring 581

2.3. Target-Organ Damage 581

**2.3.1. Central Arterial Pressure and Arterial Stiffening 581**

**2.3.2. Left Ventricular Hypertrophy 581**

**2.3.3. Albuminuria 581**

**2.3.4. Inflammatory Biomarkers 581**

3. Flowchart of Assessment of Resistant Hypertension 581

3.1. Flowchart of the Diagnostic Approach in Resistant Hypertension 581

4. Blood Pressure Measurement 582

4.1. Office Blood Pressure in Resistant Hypertension 582

4.2. Ambulatory Blood Pressure Monitoring in Resistant Hypertension 582

4.3. Home Blood Pressure Monitoring and Blood Pressure Self-Measurement 583

4.4. Measurement of Central Arterial Pressure 583

5. Target-Organ Damage 583

5.1. Introduction 583

5.2. Vascular Changes 583

5.3. Cerebral Changes 583

5.4. Cardiac Changes 583

5.5. Renal Changes 584

6. Phenotype of the Patient with Resistant Hypertension 584

6.1. Introduction 584

6.2. Phenotype of the Patient with Resistant Hypertension 584

6.3. Phenotype of Controlled and Uncontrolled Resistant Hypertension 585

**6.3.1. Pathophysiological Aspects 585**

**6.3.2. Clinical Differences 585**

**6.3.3. Prognosis 585**

6.4. Phenotype of the Patient with Refractory Hypertension 586

7. Secondary Causes of Resistant Hypertension 586

7.1. Introduction 586

7.2. Secondary Hypertension due to Non-Endocrine Causes 586

**7.2.1. Obstructive Sleep Apnea 586**

**7.2.2. Renal Parenchymal Disease 586**

**7.2.3. Renal Artery Stenosis 586**

7.3. Secondary Hypertension due to Endocrine Causes 586

**7.3.1. Primary Hyperaldosteronism 586**

**7.3.2. Pheochromocytoma 587**

**7.3.3. Hypothyroidism and Hyperthyroidism 587**

8. Non-Pharmacological Treatment 587

8.1. Weight Loss 587

8.2. Salt Restriction 588

8.3. Alcohol Intake 588

8.4. Physical Activity 589

9. Pharmacological Treatment of Resistant Hypertension 589

10. New Treatments of Resistant Hypertension 589

10.1. Introduction 589

10.2. Direct Carotid Sinus Stimulation 589

10.3. Renal Sympathetic Denervation 590

10.4. Use of Continuous Positive Airway Pressure 590

10.5. Arteriovenous Fistula 591

References 591

## 1. Definition and Epidemiology

**Coordinator:** Heitor Moreno Júnior.

**Authors:** Juan Carlos Yugar-Toledo, Heitor Moreno Júnior, Miguel Gus, Guido Bernardo Aranha Rosito, and Luiz César Nazário Scala.

### 1.1. Definition/New Concepts

Resistant hypertension (RHTN) is defined as blood pressure (BP) persistently above the recommended target values despite the use of three antihypertensive agents of different classes, including one blocker of the renin-angiotensin system (angiotensin-converting enzyme inhibitor [ACEI] or angiotensin receptor blocker [ARB]), one long-acting calcium channel blocker (CCB), and one long-acting thiazide diuretic (TZD) at maximum recommended and tolerated doses, administered with appropriate frequency and doses and with proven adherence.

Other drugs may be added if the above ones fail (aldosterone antagonists, beta-blockers, and α-methyldopa). Experts disagree on issues related to dose/potency, although the main discussion occurs around the use of chlorthalidone or hydrochlorothiazide as the main TZD.^1^

The definition above includes a subgroup of patients with RHTN whose BP is controlled with four or more antihypertensive medications, known as controlled RHTN (C-RHTN).^2 , 3^A proposal to classify the disease into C-RHTN and uncontrolled RHTN (UC-RHTN),^4^including refractory RHTN (Ref-RHTN), an extreme UC-RHTN phenotype involving use of five or more antihypertensive agents,^5^has gained space in the literature.^6 , 7^

Thus, UC-RHTN is defined by BP levels that remain above the desired level (140/90 mmHg) despite the concomitant use of four or more antihypertensive agents of different classes and a fourth drug, which is generally a mineralocorticoid receptor antagonist or a central sympathetic inhibitor ( Chart 1 ).

**Chart 1:** – Classification of resistant hypertension

	Number of antihypertensive agents	
Controlled resistant hypertension	N	Uncontrolled resistant hypertension
	6	
	5	
	4	
	3	Resistant hypertension
	2	
	1	
< 140/90	Blood pressure (mmHg)	≥ 140/90
Normotension		Hypertension

### 1.2. Control of Hypertension in Brazil and Worldwide

An analysis of 135 population studies with 1 million individuals indicated that 31.1% of the adult population is hypertensive (95% CI; 30 to 32%), with an estimated rate of 28.5 and 31.5% in countries with the highest and lowest socioeconomic status, respectively. BP control varies according to socioeconomic status, reaching 28.4% in more developed countries and only 7.7% in those with a lower degree of development.^8^In Brazil, the control rate varied from 10.4 to 35.2% in populations studied in three regions of the country.^9^

A study conducted in 291 centers in all five Brazilian regions including 2,810 patients evaluated the control rates of hypertension according to risk profile and target BP. For patients with lower risk and target levels < 140/90 mmHg, the control rate was 61.7%, while for those with high risk and target levels < 130/80 mmHg, the corresponding value was 41.8%.^10^

### 1.3. Incidence and Prevalence of Resistant Hypertension

The prevalence of RHTN among individuals with hypertension is estimated at 10 to 20% worldwide, resulting in approximately 200 million individuals with RHTN.^11^This variability is mainly due to differences in RHTN criteria and characteristics of the studied populations.

The National Health and Nutrition Examination Survey (NHANES) reported a prevalence of RHTN of about 9% in individuals with hypertension, corresponding to 12.8% of the individuals using antihypertensive agents in the US.^12^

Still, the actual prevalence of RHTN is unknown. A meta-analysis by Achelrod et al.^11^evaluating populations of individuals with treated hypertension found a prevalence of 13.72% (95% CI; 11.19 to 16.24%), according to 20 observational studies, and 16.32% (95% CI; 10.68 to 21.95%), according to four randomized controlled trials.^11^In Brazil, a multicenter study (ReHOT) including ambulatory BP monitoring (ABPM) showed a prevalence of RHTN of 11.7%.^13^

Daugherty et al.^14^analyzed the incidence of RHTN in 205,750 patients with hypertension who initiated antihypertensive treatment between 2002 and 2006. The authors found a rate of 1.9% at 1.5 years of follow-up (0.7 per 100 patients per year), leading to a 1.47 higher cardiovascular (CV) risk at 3.8 years.^14^

### 1.4. Factors Related to Resistant Hypertension

RHTN is more prevalent in elderly, obese, and African descent individuals, as well as in patients with left ventricular hypertrophy (LVH), diabetes mellitus, chronic nephropathy, metabolic syndrome, increased alcohol and/or salt intake, and sedentary lifestyle.^1 , 15 - 17^Aspects related to RHTN include the following: 1) diagnostic factors – inadequate BP measurement technique, white-coat effect;^1 , 15^2) causal factors – increased salt sensitivity, volume expansion due to excessive salt intake or chronic kidney disease (CKD), use of nonsteroidal antiinflammatory drugs, anabolic steroids, oral contraceptives, sympathomimetic agents (nasal decongestants, appetite suppressants, cocaine), chemotherapeutic agents, antidepressants, erythropoietin, immunosuppressants, alcohol;^1 , 15^3) secondary causes of hypertension, including primary hyperaldosteronism, obstructive sleep apnea (OSA), CKD, renal artery stenosis, thyroid diseases;^15^4) therapeutic factors – medications that are either inappropriate or are used in insufficient doses, medical inertia, low adherence.^16 , 17^Both systolic and diastolic hypertension may be resistant, the former being more prevalent.^1^

## 2. Prognostic Aspects

**Coordinator:** Elizabeth Silaid Muxfeldt.

**Authors:** Alexandre Alessi, Andrea Araújo Brandão, Osni Moreira Filho, and Elizabeth Silaid Muxfeldt.

### 2.1. Introduction

RHTN is associated with high CV morbidity and mortality, increasing the risk of CV events by 47% in patients affected by this condition when compared with individuals with incident hypertension.^14^

### 2.2. Office Blood Pressure and Ambulatory Blood Pressure Monitoring

True RHTN, diagnosed by ABPM, is associated with twice the CV risk compared with RHTN due to a white-coat effect.^18^Overall, the average BP measurements obtained in all three ABPM periods are strong predictors of CV risk, while office BP has shown no prognostic value.^18 , 19^Longitudinal studies have highlighted high BP during sleep and the absence of nocturnal dipping as important predictors of CV risk.^18 - 20^Prognostic importance of the nighttime BP pattern has also been shown in meta-analyses.^21^

### 2.3. Target-Organ Damage

#### 2.3.1. Central Arterial Pressure and Arterial Stiffening

Pulse wave velocity (PWV) has an independent predictive value in several subgroups of patients with hypertension.^22^Reduced arterial relaxation and elasticity have been observed in patients with RHTN compared with individuals with well-controlled hypertension, being a marker of prognosis and response to antihypertensive therapy.^23^In hypertensive patients, PWV provides additive value when incorporated into CV risk scores.^24^

#### 2.3.2. Left Ventricular Hypertrophy

The electrocardiographic diagnosis of LVH has emerged as a predictor of risk for coronary disease (Cornell index) and cerebrovascular disease (Sokolow-Lyon index), and the regression of both indices reduces the risk of CV events by 35 and 40%, respectively.^25^

#### 2.3.3. Albuminuria

Both baseline and serial changes in albuminuria have prognostic implications in RHTN. In a large prospective cohort of 531 patients with RHTN, the occurrence of moderately increased albuminuria (MIA) at baseline was an independent predictor of composite events and all-cause mortality.^26^A later analysis by the same group, this time including 1,048 patients, showed that MIA increased by 40% the risk of fatal and nonfatal CV events and all-cause mortality.^27^

During follow-up, the persistence of MIA at 2 years was a risk factor for CV events, while persistent normoalbuminuria emerged as a protective factor.^26^Another cohort of 143 patients with RHTN assessed at baseline and after 6 years of follow-up showed that the development or persistence of MIA was associated with an increased risk of CV events. In contrast, the persistence of normoalbuminuria or regression of MIA was associated with a lower risk of major events.^28^

#### 2.3.4. Inflammatory Biomarkers

Elevated C-reactive protein is an independent predictor of coronary and cerebrovascular disease, and a more important marker in patients with RHTN who are younger, obese, and have uncontrolled ABPM and a non-dipping pattern (absent or attenuated nocturnal decline).^29^

## 3. Flowchart of Assessment of Resistant Hypertension

**Coordinator:** Audes Diógenes de Magalhães Feitosa.

**Authors:** Oswaldo Passarelli Júnior, Dilma do Socorro Moraes de Souza, and Audes Diógenes de Magalhães Feitosa.

### 3.1. Flowchart of the Diagnostic Approach in Resistant Hypertension

On clinical suspicion of RHTN, diagnostic confirmation is required, and the first step in the investigation is the exclusion of causes of pseudoresistance, such as lack of treatment adherence (pharmacological and non-pharmacological), inadequate dosing, improper BP measurement technique, and white-coat effect^1^( Figure 1 ). Lack of BP control should be confirmed by ABPM and home blood pressure monitoring (HBPM).^30 - 32^

**Figure 1:** – *Flowchart of the evaluation of resistant hypertension. ABPM: ambulatory blood pressure monitoring; HBPM: home blood pressure monitoring; Na^+^: sodium; BP: blood pressure.*

Once pseudoresistance is excluded, the occurrence of RHTN is confirmed and a diagnostic investigation should be initiated with specific tests, according to recommendations of hypertension guidelines regarding the involvement of target-organ damage (TOD) and secondary hypertension.^33 , 34^The occurrence of associated comorbidities should be evaluated with specialized tests according to clinical suspicion.

Out-of-office BP measurement is fundamental since such readings are usually higher than those measured at home, reflecting the frequent occurrence of the white-coat effect in this population. Treatment adherence is always challenging, especially in public centers.

Patient-related problems that may occur include rejection to the excessive number of medications in complex dosing (excessive doses and tablets), medication side effects, sociocultural issues and lack of knowledge of the natural history of the disease, as well as other problems related to the physician, including poor doctor-patient relationship, non-synergistic dosing or wrong doses and omission or lack of knowledge in the investigation of treatable secondary causes. A potential problem related to health care services is difficulty in access to physicians, medications, and complementary tests.

All these factors hinder the adherence to pharmacological and non-pharmacological treatment and must, therefore, be verified and circumvented.

Salt intake should always be verified, if possible with a 24-hour urinary sodium measurement, as intake is often excessive due to the consumption of processed foods and lack of knowledge by the patients about excessive salt intake.

Treatment should be optimized, preferably with the same physician and for a minimum of 6 months to strengthen the doctor-patient relationship. Added to that are regular recommendations regarding healthy lifestyle habits and continuous verification of treatment adherence, with synergistic dosing schedules and appropriate medication adjustments, respecting the occurrence of comorbidities indicating or contraindicating certain antihypertensive drug classes.

## 4. Blood Pressure Measurement

**Coordinator:** Celso Amodeo.

**Authors:** Weimar Kunz Sebba Barroso, Marco Antônio Mota Gomes, Annelise Machado Gomes de Paiva, and Eduardo Costa Duarte Barbosa.

### 4.1. Office Blood Pressure in Resistant Hypertension

Although not diagnostic of RHTN, office BP should be verified, and the measurement procedure should follow the guidelines of the 7^th^Brazilian Guideline of Arterial Hypertension.^33^The BP can be measured with a manual, semiautomatic, or automatic sphygmomanometer. Several measurements are recommended, with the patient sitting in a calm and comfortable environment to improve reproducibility and bring the values obtained in the office close to those obtained on ABPM during daytime.

Consideration must be given to the occurrence of the white-coat effect, a phenomenon involving two situations. The first is a white-coat hypertension, in which BP is elevated in isolated office measurements but normal during ABPM or HBPM. The second is a white-coat effect, which is characterized by increased office BP in relation to the mean BP during daytime in the ABPM or the weekly average HBPM, without changing the diagnosis of hypertension or normotension.^35^

These two situations can lead to a false diagnosis of RHTN, resulting in unnecessary test requests and medication use. White-coat hypertension may be referred to as a cause of pseudo-resistant hypertension.^36^

### 4.2. Ambulatory Blood Pressure Monitoring in Resistant Hypertension

This test is necessary to rule out the hypothesis of white-coat hypertension, which falsely suggests RHTN.^37^The diagnosis is confirmed when the mean BP during daytime and over 24 hours is below 135/85 mmHg and 130/80 mmHg, respectively. Compared with casual BP measurements, the values obtained are more strongly related to the risks arising from hypertension, especially during ABPM evaluation, when an absence or attenuation of the BP reduction during sleep is identified, along with an increase in the difference between systolic and diastolic BP.^37^ Chart 2 presents the main applicability in hypertension of the ABPM, a fundamental test in RHTN evaluation, diagnosis, and follow-up.

**Chart 2:** – Key information obtained from ambulatory blood pressure monitoring

Multiple measurements over an observation period
Blood pressure assessment during daytime
Correlation of daytime measurements with activities and symptoms
Blood pressure assessment during sleep
Possibility of correlating blood pressure variability with symptoms, activities, and medications
Complement to the patient's diagnosis and prognosis
Evaluation of the antihypertensive effect

### 4.3. Home Blood Pressure Monitoring and Blood Pressure Self-Measurement

Home BP measurements are more accurate than casual BP measurements and offer a better prediction of risk for CV outcomes, contributing to greater adherence to drug treatment.^35 , 38 , 39^In this context, HBPM and BP self-measurement (BPSM) are viable and effective alternatives for proper diagnosis and improved adherence.^40 , 41^

### 4.4. Measurement of Central Arterial Pressure

Arterial stiffness is recognized as an important prognostic index and potential therapeutic target in patients with hypertension. As a result, central systolic blood pressure (cSBP) and PWV have been recently investigated in a population of patients with RHTN.^42^The mean age of the population was 58.7 ± 15.3 years, and 65% (n = 53) were women. Brachial and central blood pressures were elevated in all patients. Additionally, the PWV value was higher than the reference value for age, and the difference was statistically higher for PWV in women.

Another study^23^analyzing associations between RHTN and arterial stiffness has shown that patients with RHTN have increased vascular stiffness compared with patients with well-controlled hypertension. PWV increased with arterial stiffness and correlated with BP levels, justifying the need for adequate BP control.

## 5. Target-Organ Damage

**Coordinator:** Roberto Dischinger Miranda.

**Authors:** José Fernando Vilela-Martin, Juan Carlos Yugar-Toledo, Wilson Nadruz Júnior, and Cibele Isaac Saad Rodrigues.

### 5.1. Introduction

Both C-RHTN and UC-RHTN are associated with a higher prevalence of TOD and higher CV risk and mortality compared with controlled hypertension.^43 - 45^Therefore, the investigation of TOD in RHTN is fundamental to complement the risk stratification and establish the prognosis.^44^

### 5.2. Vascular Changes

Patients with RHTN present structural and functional vascular changes resulting not only from uncontrolled hypertension but also from early vascular aging. This is a complex process involving biochemical, enzymatic, and cellular changes that modify the function and structure of the artery, culminating in early and progressive degeneration of the arterial health.^43 - 47^

Pathophysiological mechanisms include increased oxidative stress, endothelial dysfunction, vascular remodeling, smooth muscle cell hypertrophy, increased arterial stiffness due to changes in collagen/elastin distribution, vascular inflammation, and increased expression of inflammatory mediators and matrix repair metalloproteinases, in addition to increased advanced glycation end-products and parietal calcification.^48 , 49^

The molecular mechanisms of vascular aging include genetic alterations in segments involved in DNA protection and repair^50^and mitochondrial metabolic activity.^51^

In the microcirculation, endothelial dysfunction promotes vasoconstriction, eutrophic remodeling (increased media/lumen [M/L] ratio without external changes), decreased vasodilatory reserve and vascular rarefaction, the latter evaluated by *in vivo* capillaroscopy,^52^gluteus biopsy, or yet, measurement of the M/L ratio with laser Doppler flowmetry of retinal arteries^53^and optical videomicroscopy. In large arteries, parietal remodeling leads to increased arterial stiffness.^49 , 54 - 56^

Arterial stiffness is estimated by carotid-femoral PWV (c-f PWV) and calculation of the augmentation index (AIx) by applanation tonometry.^57 , 58^These changes in arterial stiffness hemodynamic parameters and cellular biomarkers are associated with increased morbidity and mortality.^59 , 60^

The macrovascular involvement is further characterized by carotid, cerebral, coronary, and peripheral atherosclerotic diseases.^61 , 62^

### 5.3. Cerebral Changes

The cerebrovascular involvement in RHTN is subtle and insidious. Microscopic white matter lesions begin early and may progress irreversibly, leading to cognitive impairment and progression to vascular dementia.^63 , 64^

Patients with RHTN have a higher risk of cerebral infarction and transient cerebral ischemia, a fact that has been pointed out by the Kaiser Permanente^16^and REGARDS studies,^65^which showed risk increases of 17 and 14%, respectively. Atherosclerosis of the carotid and small cerebral vessels is responsible for ischemic and thromboembolic phenomena. Retinal artery occlusion is a marker of small vessel injury and has been associated with an increased risk of cerebral events.^66^

Uncontrolled hypertension is the leading cause of hemorrhagic stroke. Patients with RHTN have microangiopathy (Charcot-Bouchard aneurysms), which affect the penetrating arteries in the brain and cause intraparenchymal hemorrhage.^67^

Changes in large artery stiffness are also associated with increased occurrence of microvascular changes and a greater predisposition to cerebrovascular events.^68^

### 5.4. Cardiac Changes

Several cardiac changes may be observed in patients with RHTN, including LVH, left ventricular diastolic dysfunction (LVDD), and myocardial ischemia.^69^LVH is an independent predictor of heart failure, coronary artery disease (CAD), arrhythmias, and stroke.^70^

In Brazil, the prevalence of LVH assessed by echocardiography in patients with RHTN ranges from 68 to 87%,^71 , 72^with concentric LVH being the most common geometric pattern in these individuals.^72 , 73^

LVDD predisposes to cardiovascular events and heart failure, regardless of cardiac mass and BP levels.^74^The exact prevalence of LVDD in patients with RHTN is uncertain, but the strong association between this condition and LVH^74^suggests that LVDD is very frequent in this population. About one third of the patients with RHTN are diagnosed with CAD.^71^However, even in the absence of overt CAD, up to 28% of the patients with RHTN have myocardial ischemia,^72^which may result from decreased coronary reserve and increased myocardial oxygen consumption, particularly in patients with LVH, and increased arterial stiffness.^70 , 74^

### 5.5. Renal Changes

The association between RHTN and CKD is well established and may be causal or consequential. The anatomopathological substrate is hypertensive nephrosclerosis, resulting from hemodynamic abnormalities (glomerular hyperfiltration and hypertrophy), culminating in glomerulosclerosis. Nephrosclerosis (erroneously termed “benign”) is characterized by arteriosclerosis and arteriolosclerosis, hyalinosis, tubulointerstitial lesions, global glomerulosclerosis, and focal segmental glomerulosclerosis.

Known risk factors for CKD progression include age > 50 years, male sex, genetic predisposition, family history, African descent, hypertension duration and stage, low socioeconomic status, intensity of albuminuria, degree of renal dysfunction, dyslipidemia, obesity, diabetes, lifestyle habits (diet with excessive salt and/or protein, smoking), and use of nephrotoxic substances, among others.^75^Albuminuria and reduced estimated glomerular filtration rate (eGFR) identify patients at high CV and renal risks, and the reduction in albuminuria may be a therapeutic objective in RHTN.^26 - 28^

Recommended tests for evaluation and follow-up of renal damage include urinalysis, serum creatinine for eGFR calculation using the equations MDRD or CKD-EPI, available at http://ckdepi.org/equations/gfr-calculator/, renal and urinary tract ultrasonography, and calculation of the albuminuria or urinary protein/creatinine ratio for CKD staging^75^( Figure 2 ).

**Figure 2:** – *Prognosis of chronic kidney disease according to degrees of albuminuria and decline in estimated GFR.^76^Green: low risk; yellow: moderate risk; orange: high risk; red: very high risk.*

## 6. Phenotype of the Patient with Resistant Hypertension

**Coordinator:** Luciano Ferreira Drager.

**Authors:** Heitor Moreno Júnior, Juan Carlos Yugar-Toledo, and Luiz Aparecido Bortolotto.

### 6.1. Introduction

This section describes initially the characteristics that distinguish patients with RHTN from those with non-resistant hypertension. Subsequently, it discusses the differences between patients with C-RHTN and UC-RHTN, and finally, addresses the approach to the extreme phenotype of the RHTN patients, i.e., patients with refractory hypertension.

### 6.2. Phenotype of the Patient with Resistant Hypertension

Patients with RHTN often present some characteristics that distinguish them from those with non-resistant hypertension, including older age, obesity, a profile of increased salt intake, CKD, diabetes, presence of TODs such as LVH, female sex, and african descent.^1^The Brazilian multicenter study ReHOT has shown that diabetes, prior stroke, and BP at study entry ≥ 180/110 mmHg (hypertension stage 3) were independent predictors of true resistance.^13^While some of these characteristics are intuitive, others, including the female sex, still lack well-defined rationales in predicting RHTN.

### 6.3. Phenotype of Controlled and Uncontrolled Resistant Hypertension

#### 6.3.1. Pathophysiological Aspects

C-RHTN shows greater dependence on volume status than UC-RHTN, due to critical persistence of water retention, increased sodium sensitivity, hyperaldosteronism, and renal dysfunction. Additionally, these individuals present increased plasma volume expansion measured by thoracic bioimpedance,^77^higher plasma and urinary aldosterone concentrations, suppression of renin activity,^78^high plasma aldosterone/renin ratio (ARR), and increased levels of atrial natriuretic peptide (ANP) and brain natriuretic peptide (BNP).^79 - 83^This relationship between increased volume and pressure is the pathophysiological basis demonstrated in several studies^81 , 84 , 85^and justifies the use of diuretics in patients with C-RHTN.^86 , 87^

In contrast, patients with UC-RHTN often have sympathetic hyperactivity, evidenced by increased (24-hour) urinary metanephrines and resting heart rate, reduced 24-hour variability (spectral analysis), in addition to increased vascular stiffness (increased PWV).^88 , 89^These markers of increased sympathetic activity, together with other factors linked to hyperaldosteronism,^78 , 90 - 92^are related to mechanisms that maintain high BP even with administration of four or more antihypertensive agents, characterizing UC-RHTN. Higher PWV values reflect exacerbated arterial stiffness,^4^while elevated levels of cytokines, including tumor necrosis factor-alpha (TNF-α),^48 , 56 , 93^probably indicate vascular damage in patients with RHTN.^49^

Other factors and mechanisms, such as age, obesity, OSA,^4 , 94 , 95^African descent, adipokine deregulation,^96^endothelial dysfunction, and increased activity of metalloproteinases-2, metalloproteinases-9 and adhesion molecules^97 - 99^are also involved in this process.

Genetic polymorphisms, especially those involving the renin-angiotensin-aldosterone system and the endothelial nitric oxide synthase (eNOS), have been correlated to RHTN^100 , 101^( Figure 3 ). However, large studies conveniently characterized in individuals with the disease are needed to define the importance of genetics in this group of patients.

**Figure 3:** – *Predominant pathophysiological mechanisms in controlled (C-RH) and uncontrolled (U-RH) resistant hypertension. Refractory hypertension (uncontrolled with five or more medications) is included in the U-RH group.*

#### 6.3.2. Clinical Differences

In 2011, Martins et al. published a comparative study in patients with C-RH and UC-RHTN^4^specifically assessing biological factors contributing to resistance to antihypertensive agents. Body mass index (BMI), arterial stiffness (PWV), left ventricular mass index (LVMI), and plasma aldosterone concentration (PAC) were higher in the UC-RHTN group when compared with the C-RHTN group. Additionally, the authors demonstrated using multivariate analysis that PWV was dependent on age in both groups, although this influence was more pronounced in patients with UC-RHTN. They also showed that the UC-RHTN group had higher values of carotid intima-media thickness (cIMT) and PWV.^102^Finally, the drop in nocturnal BP (dipping pattern) was less pronounced in the UC-RHTN group.^103^

#### 6.3.3. Prognosis

Pierdomenico et al.^104^evaluated CV outcomes in subjects with C-RHTN and UC-RHTN. The occurrence of fatal and nonfatal CV events was investigated in 340 patients with C-RHtN (BP < 140/90 mmHg or daytime BP < 135/85 mmHg) and 130 patients with UC-RHTN (BP ≥ 140 or 90 mmHg and daytime BP > 135 or 85 mmHg). During follow-up (4.98 ± 2.9 years), the event rates per 100 patients/year were 0.87 and 4.1, respectively. These data also show that patients with UC-RHTN have a greater risk of CAD, stroke, arterial disease, congestive heart failure (CHF), kidney disease, and all-cause death compared with patients with C-RHTN.

## 6.4. Phenotype of the Patient with Refractory Hypertension

Refractory hypertension appears to be an extreme phenotype of patients with RHTN. Recently, phenotypic characterization has shown that these patients are younger than those with RHTN in general, are more commonly women, have a higher frequency of heart failure, and have particularly higher sympathetic activity than patients with RHTN.^5^These findings are important pillars for the pathophysiology of refractoriness, potentially constituting a therapeutic target for procedures such as renal denervation. Studies in this area are currently under development.

## 7. Secondary Causes of Resistant Hypertension

**Coordinator:** Fernanda Marciano Consolim-Colombo

**Authors:** Márcio Gonçalves de Sousa, Flávio Antonio de Oliveira Borelli, Cibele Isaac Saad Rodrigues, and Fernanda Marciano Consolim-Colombo.

### 7.1. Introduction

Secondary hypertension (SecHTN) is defined as increased BP due to an identifiable cause.^33 , 105^Patients with RHTN should be investigated for the most prevalent causes of “non-endocrine” and “endocrine” SecHTN after exclusion of use of medications that may interfere with BP values: antiinflammatory drugs, glucocorticoids, nasal decongestants, appetite suppressants, antidepressants, immunosuppressants, erythropoietin, contraceptives, and illicit drugs.^33 , 105^

### 7.2. Secondary Hypertension due to Non-Endocrine Causes

#### 7.2.1. Obstructive Sleep Apnea

Defined as a total or partial cessation of respiratory flow during sleep, this syndrome promotes oxyhemoglobin desaturation and microarousals during sleep. OSA is estimated to have a prevalence of 17%^106^among American adults and 30% among hypertensive individuals and may affect 60 to 80% of the patients with RHTN.^94^A recent meta-analysis^107^has concluded that the presence of OSA is related to a higher risk of RHTN.^107^

Activation of the sympathetic nervous system and humoral abnormalities are responsible for changes in vascular endothelial integrity, and their consequences in patients with OSA include increased BP, development of atherosclerotic disease, and cardiac arrhythmias, among others.^108^Clinical suspicion can be verified with the Berlin questionnaire.^109^

The diagnosis is established with polysomnography, which records apnea/hypopnea indices greater than five events/hour.

Treatment should include recommendations on sleep hygiene and weight loss, among others. For airway clearance, the use of equipment producing continuous positive airway pressure (CPAP) is the most recommended. However, the impact of this treatment on reducing BP values is still debatable.^110 , 111^

#### 7.2.2. Renal Parenchymal Disease

Renal parenchymal disease (RPD) is one of the most prevalent causes of SecHTN. The diagnosis of this condition is relatively simple since the assessment of renal function is part of the routine approach in patients with hypertension. Patients on dialysis and renal transplant recipients have a high prevalence of hypertension, and CV events are responsible for high morbidity and mortality in this population.^112^

The progression of renal dysfunction in patients with RPD is directly related to BP values, and target BP levels should be achieved to reduce CV morbidity and mortality. In patients with RPD and renal transplant recipients, ACEIs and angiotensin-II receptor blockers have been shown to offer renal protection additional to that obtained by BP reduction, and are, therefore, the preferred medications.^33 , 105 , 113^

#### 7.2.3. Renal Artery Stenosis

Renovascular disease is a term used to define renal artery involvement by different pathologies, including atherosclerotic disease, fibromuscular dysplasia, and vasculitis, which can lead to arterial obstruction. Usually, no symptoms are associated with mild arterial obstruction. However, with obstructions affecting more than 70% of the artery, severe hypertension and even ischemic nephropathy may occur.

Renal artery stenosis (RAS) of atherosclerotic origin is present in 12.5% of the patients with RHTN older than 50 years of age.^114^The diagnosis should always be determined, but the treatment of this condition is still much discussed in the literature.^115 , 116^Adequate BP control and interruption of progressive renal function deterioration are the primary treatment goals in these patients. To achieve that, two therapeutic possibilities are available for this population: clinical and interventional (surgical or percutaneous, with or without stent implantation).

Interventions are recommended for patients with RHTN or accelerated hypertension with progressive loss of renal function, bilateral RAS or stenosis in a “single” kidney, or with severe complications (CHF and recurrent acute pulmonary edema).^33 , 115 , 116^

Other potential surgical indications include total renal artery obstruction, large arteriovenous fistulas, aortic lesion encompassing the renal arteries, and failure in clinical or endovascular treatment.^117^

## 7.3. Secondary Hypertension due to Endocrine Causes

### 7.3.1. Primary Hyperaldosteronism

Considered in the past to be a rare type of SecHTN (with a prevalence of about 1%), hyperaldosteronism is currently believed to occur in up to 22% of the cases in populations with RHTN.^118 , 119^The most frequent cause of hyperaldosteronism is adrenal adenoma, while unilateral or bilateral hyperplasia is less frequently detected. Carcinomas (albeit infrequent) and genetic forms of the disease may also be responsible for the occurrence of hyperaldosteronism.

Aldosterone, through activation of mineralocorticoid receptors, is related to insulin resistance and endothelial dysfunction and, consequently, participates in the development of metabolic syndrome and CV and renal lesions associated with RHTN. Thus, mineralocorticoid receptor blockade improves endothelial dysfunction and contributes to a better response to RHTN and TOD therapies.^118 , 119^

During the diagnostic evaluation, all patients with RHTN (not only those with hypokalemia) should be evaluated for the occurrence of hyperaldosteronism.^33^Screening should include the assessment of the plasma aldosterone concentration (PAC expressed in ng/dL) to plasma renin activity (PRA expressed in ng/mL/hr) called aldosterone/renin ratio (ARR). This method has excellent sensitivity but may yield false-positive results. Therefore, adoption of the minimum PAC and PRA values of 15 ng/dL and 0.5 ng/mL/h, respectively, are recommended. An ARR ≥ 100 establishes the diagnosis of hyperaldosteronism, while values < 20 to 30 indicate a low probability of the disease, and values in between detect “individuals potentially affected” by this condition.^120^In the latter case, tests assessing the renin-aldosterone axis (saline infusion test, walking, use of diuretics) may be performed.

Tomography or magnetic resonance imaging is used for imaging identification of adrenal adenomas or hyperplasia. The absence of a visible tumor on tomography does not exclude the presence of a microadenoma, hence the importance of searching for excessive aldosterone production. Functional images, obtained by adrenal scintigraphy, may be useful in detecting adenomas and may differentiate them from nodular hyperplasia in up to 90% of the cases. Adrenal vein blood sampling can be used to confirm lateralization in aldosterone secretion and the presence of unilateral adenoma.^120 , 121^

In terms of treatment, unilateral resection usually corrects excessive aldosterone production and potassium loss in unilateral adenomas. The BP response to surgical treatment varies. Cases of hyperplasia benefit from aldosterone receptor blockade.^121^

### 7.3.2. Pheochromocytoma

Pheochromocytoma is a rare neuroendocrine tumor that originates from chromaffin cells (cells producing catecholamines). The most common clinical manifestation of this condition is elevated BP, and the disease may arise from the adrenal medulla or extra-adrenal paraganglia (paragangliomas). Clinical exacerbation peaks between the third and fourth decades of life, but 10% of the cases arise in childhood.

These tumors may be sporadic or associated with genetic syndromes.^122 , 123^They are usually unilateral; however, in familial syndromes, they may be bilateral, multiple, or extra-adrenal, and benign or malignant (5 to 26% of the cases). This etiology should be investigated in all patients presenting with RHTN and/or symptoms or signs suggestive of hyperadrenergic spells. Paroxysmal hypertension occurs in 30% of the cases, triggered by regular physical activity, exercises with increased intensity, surgical procedures, and use of certain substances such as tricyclic antidepressants, histamine, and opioids. Paroxysms may be accompanied by headache (60 to 90%), sweating (55 to 75%), and palpitations (50 to 70%). Symptoms of heart failure and electrocardiographic abnormalities may indicate myocarditis induced by catecholamine excess.

At diagnosis, measurement of metanephrines (catecholamine metabolites) in plasma and 24-hour urine has higher sensitivity and specificity than direct catecholamine measurement. When laboratory tests are not elucidative, clonidine suppression test may be performed (administration of clonidine 0.2 mg and measurement of catecholamines 1 hour before and 2 hours after the medication).

For a topographic diagnosis of the tumors and, eventually, the metastases, the recommended imaging methods are computed tomography and magnetic resonance imaging, both of which have sensitivity close to 100% for adrenal tumors. Whole-body 131 or 123 metaiodobenzylguanidine (MIBG) has sensitivity of 56 to 85% (malignant tumors) and high specificity. Octreoscan, bone mapping, and PET scan (with different markers) can be decisive when previous localization tests are negative or in the investigation of malignant disease.

Treatment is surgical. However, in preoperative or chronic medication therapy, alpha-blockers (prazosin, doxazosin, and dibenzyline) are initially used, combined or not with other agents such as beta-blockers (after effective alpha blockade), ACEIs, and CCBs. Control of BP levels and volume replacement are recommended before the surgical intervention.^124^Sodium nitroprusside can be used in acute crises and during surgery.^124^

### 7.3.3. Hypothyroidism and Hyperthyroidism

Hypertension may affect 40% of the patients with thyroid disorders, while correction of the glandular dysfunction usually results in BP control.^125^If BP levels remain high after correction of the hypothyroidism or hyperthyroidism, use of antihypertensive drugs is indicated.^32 , 126^

Causes of SecH in patients with RHTN are summarized in Table 1 .

**Table 1:** – Prevalence, clinical findings, and additional tests for the most common causes of secondary hypertension in patients with resistant hypertension

Secondary cause	Overall prevalence	Prevalence in RHTN	Clinical findings	Diagnostic investigation
Obstructive sleep apnea^94,107,109^	> 5 to 15%	> 30%	Snoring, daytime sleepiness, morning headache, metabolic syndrome	Berlin questionnaire, STOP-Bang questionnaire, Epworth sleepiness scale, polysomnography (gold standard) or home polysomnography with five or more episodes of sleep apnea and/or hypopnea per hour of sleep
Renal parenchymal disease^113^	1.6 to 8%	2 to 10%	Edema, anorexia, nocturia, fatigue, anemia, increased urea and creatinine, changes in urinary sediment	Urinalysis (low density, glomerular hematuria or albuminuria), calculation of estimated GFR, renal US, screening for albuminuria and protein/creatinine ratio in random urine sample
Renal artery stenosis^115,116^	1 to 8%	2.5 to 20%	Abdominal murmur, acute pulmonary edema, impaired renal function by RAAS blockers, asymmetric kidneys	Screening: renal artery Doppler US (operator dependent) and/or renogram with or without captopril, magnetic resonance angiography, computed tomography, conventional renal arteriography (gold standard)
Primary hyperaldosteronism^119-121^	1.4 to 10%	6 to 23%	Mostly asymptomatic RH hypokalemia (not required and unusual) Incidental adrenal nodule	ARR > 30 in the absence of aldosterone antagonists. Confirmatory tests (suppression with fludrocortisone or saline infusion) Imaging tests: thin-slice helical computed tomography (preferred) or resonance magnetic imaging
Thyroid diseases^32^Hypothyroidism	1 to 2%	1 to 3%	Fatigue, weight gain, hair loss, systolic hypertension, muscle weakness.	TSH and free T4
Hyperthyroidism			Heat intolerance, weight loss, diastolic hypertension, palpitations, exophthalmos, tremors, tachycardia	
Cushing’s syndrome^32^	0.5%	< 1%	Weight gain, fatigue, hirsutism, amenorrhea, “moon facies,” “buffalo hump,” purple striae, central obesity, hypokalemia	Salivary cortisol 24-hour urinary cortisol Morning cortisol (8 AM) and 8 hours after administration of dexamethasone (1 mg) at 12 AM. Magnetic resonance
Pheochromocytoma^127,128^	0.2 to 0.5%	< 1%	Episodic, labile or resistant hypertension, episodic headache, profuse sweating and palpitations, pallor	Free plasma and/or 24-hour urinary metanephrines (values twice or thrice above the normal), 24-hour plasma and/or urinary catecholamines and/or computed tomography and magnetic resonance
Coarctation of aorta^129^	< 1%	< 1%	SBP/DBP difference > 20/10 mmHg between upper and lower limbs; ejection murmur in the interscapular region	Lower rib notching on chest X-ray, screening with Doppler echocardiography, magnetic resonance imaging or thoracic aorta angiography

*Adapted from Rimoldi SF et al.^105^PA/PRA: plasma aldosterone/plasma renin activity; RHTN: resistant hypertension; DBP: diastolic blood pressure; SBP: systolic blood pressure; GFR: glomerular filtration rate; RAAS: renin-angiotensin-aldosterone system; US: ultrasonography.*

## 8. Non-Pharmacological Treatment

**Coordinator:** Sérgio Emanuel Kaiser.

**Authors:** Gil Fernando Salles, Maria de Fátima de Azevedo, and Lucélia Batista Neves Cunha Magalhães.

### 8.1. Weight Loss

Several mechanisms contribute to maintain high BP in obese patients with hypertension, including OSA, sympathetic hyperactivity, endothelial dysfunction, and modification of the intestinal microbiota – all these factors can promote an inflammatory phenotype and perpetuate a vicious cycle.^130^Patients with BMI ≥ 30 kg/m^2^are 50% more likely to have uncontrolled BP than those with normal BMI (< 25 kg/m^2^).^131^A BMI > 40 kg/m^2^triples the chances of the requirement of multiple drugs for BP control.^132^

A weight loss of 10 kg is associated with mean reductions of 6.0 mmHg in systolic BP and 4.0 mmHg in diastolic BP.^133^Surprisingly, there is no consistent evidence on the effect of diet-induced weight loss in patients with RHTN, but this recommendation meets the common sense and the evidence available in other subgroups. There are also no data on the effect of bariatric surgery on BP in this subgroup. A recent randomized trial showed a reduction of at least 30% in the number of antihypertensive drugs in 84% of operated patients, compared with 12.4% in the clinically treated group.^134^

### 8.2. Salt Restriction

Control of salt intake is especially effective in the elderly, in individuals of African descent, and in those with decreased glomerular filtration.^135^These situations restrict the ability of water and sodium excretion by the kidneys, and BP becomes more dependent on volume variations. Not surprisingly, sodium sensitivity and volume overload account for the primary pathophysiological mechanism in most cases of RHTN.^136^A systematic review and meta-analysis involving 34 studies with 3,230 participants on the effect of long-term reduction in sodium intake revealed a decrease in systolic BP of 5.8 mmHg (2.5 to 9.2; p = 0.001) associated with a decrease in urinary sodium excretion of up to 100 mmol in 24 h, which corresponds to a reduction in salt intake of approximately 6 g/day.^137^In patients with RHTN, a low-sodium diet with 2.5 g of salt daily reduced BP by up to 23.0/9.0 mmHg, clearly demonstrating the efficacy of this measure, despite the possibility of compromising the long-term adherence to such markedly restricted salt consumption.^79^

### 8.3. Alcohol Intake

Due to the direct relationship between the amount of alcohol consumed and BP levels, excessive alcohol consumption contributes significantly to hinder BP control.^138^After all, daily consumption of more than two drinks (about 24 g/day) is associated with increased BP levels.^139^A recent meta-analysis of 36 studies with 2865 participants revealed that a 50% reduction in daily alcohol intake among consumers of six or more drinks (72 g) led to a decrease of 5.50 mmHg in systolic BP (95% CI; 6.70 to 4.30) and 3.97 mmHg in diastolic BP (95% CI; 4.70 to 3.25).^140^No studies have been published on patients with RHTN; however, based on available information, daily alcohol consumption is recommended to be restricted to less than two standard drinks (about 24 g) or even interrupted.

### 8.4. Physical Activity

Despite having been evaluated only in small groups of patients with RHTN, physical activity is probably as much – or even more – beneficial in these individuals compared with those with non-resistant hypertension.^40 , 141^Regular aerobic exercise decreases office and ambulatory BP in patients with RHTN^142 - 145^and attenuates the characteristic neurohumoral activation.^146^Despite the lack of studies on resistance exercise in this subgroup, it is assumed that there is at least an advantage similar to that observed in patients with non-resistant hypertension.^147^Furthermore, the improved cardiorespiratory capacity obtained with physical activity appears to reduce mortality in patients with RHTN.^148^Therefore, this category of patients should be encouraged to perform regular physical activity of moderate intensity under proper supervision. In patients with very high BP (systolic BP ≥ 180 mmHg or diastolic BP ≥ 110 mmHg), physical activity should be delayed until the optimization of pharmacological treatment promotes BP reduction.^40 , 141^

## 9. Pharmacological Treatment of Resistant Hypertension

**Coordinator:** Rui Manoel dos Santos Póvoa.

**Authors:** Marcus Vinícius Bolívar Malachias, Armando da Rocha Nogueira, and Paulo César Brandão Veiga Jardim.

The objective of pharmacological treatment in RHTN is to identify the causes of lack of control and find the best combination of drugs, aiming at achieving the target BP with few adverse effects and greater adherence.

In general, triple treatment optimization is attempted with preferred drugs, namely, ACEIs or ARBs, dihydropyridine CCBs, and TZDs.^33 , 149^

Because they are better tolerated, ACEIs or ARBs must be increased to maximum doses in RHTN. Long-acting, higher potency TZDs, such as chlorthalidone instead of hydrochlorothiazide, should be used at appropriate doses for volume control, from 12.5 to 50 mg in a single dose in the morning.^1 , 33 , 40 , 150^Indapamide is a second TZD option in RHTN.^150^Furosemide should be used in cases of CKD with a eGFR of 30 mL/min or less.^1 , 33^In RHTN, CCB should preferably be taken at night to alternate the peaks of action of the antihypertensive drugs.^40^

Intolerance to CCBs due to side effects is often one of the causes of treatment resistance. In such cases, lipophilic CCBs (manidipine, lercanidipine, manidipine) or levamlodipine, at low doses, may be attempted or, in selected cases, a non-dihydropyridine CCB such as diltiazem and verapamil.^33^If a CCB cannot be used, introduction of a beta-blocker may be considered, preferably one with vasodilatory action, such as nebivolol or carvedilol.^33 , 151^Beta-blockers may also be considered in association with one or more preferred antihypertensive drugs – ACEI or ARB, TZD, CCB – in special conditions such as heart failure, CAD, and increased basal heart rate, among others.^33 , 150 , 151^

Failure to reach the target BP with triple therapy requires the use of a fourth drug, which current preferred option is spironolactone, 25 to 50 mg daily.^13 , 152 - 154^In cases of intolerance to spironolactone, which main adverse effect is gynecomastia in men, 12.5 mg daily may be attempted. As eplerenone is not available in our country, if intolerance to spironolactone persists even at low doses, replacement with a central sympatholytic agent should be considered, preferably clonidine, between 0.100 and 0.200 mg twice daily,^152^or a potassium-sparing diuretic, preferably amiloride (only available sparingly in our country in compounded formulations), from 10 to 20 mg;^155^or a beta-blocker, preferably with vasodilatory action, if not yet used;^40^or an alpha-blocker, preferably doxazosin 1 to 16 mg in one (nighttime) or two daily doses.^33 , 40 , 155^

All these antihypertensive agents may be used in combination when necessary for BP control.^33^When no control is obtained with the addition of the fourth drug or combinations of the following options, a direct vasodilator must be used, preferably hydralazine, at doses between 50 and 150 mg administered twice or thrice daily.^40^Due to frequent adverse effects, the vasodilator minoxidil should be reserved for situations of extreme resistance when all previous alternatives fail^40 , 150^( Figure 4 ).

**Figure 4:** – *Flowchart of RH treatment. CCB: calcium channel blocker; ARB: angiotensin receptor blocker; HR: heart rate; ACEI: angiotensin-converting enzyme inhibitor; CKD: chronic kidney disease; BP: blood pressure.*

In RHTN treatment, attention must be given to possible adverse effects of each drug used, along with their possible interactions.

## 10. New Treatments of Resistant Hypertension

**Coordinator:** Luiz Aparecido Bortolotto.

**Authors:** Luiz Aparecido Bortolotto, Luciano Ferreira Drager, and Thiago de Souza Veiga Jardim

### 10.1. Introduction

In recent years, new types of interventional treatment have been evaluated in patients with RHTN, including:

### 10.2. Direct Carotid Sinus Stimulation

Stimulation of carotid baroreceptors increases their activity and, consequently, reduces sympathetic flow, resulting in decreased BP.^156^Interventions promoting this stimulation have been used to treat patients with RHTN lacking response to clinical treatment.^156 - 159^Baroreflex activation therapy (BAT) is a surgical procedure in which electrodes are surgically implanted on the external portion of the carotid sinus unilaterally or bilaterally.^157 , 159^BAT has shown significant reductions in BP persisting for up to 3 years in randomized controlled trials.^157 , 159^However, this procedure is invasive and expensive and is associated with side effects, limiting its indication in clinical practice.^156 , 159^Another form of stimulation is the amplification of the endovascular baroreflex (implantation of an expandable device within the carotid artery), which has shown promising results with greater safety in controlling BP in RHTN.^156^These procedures are not available in Brazil.

### 10.3. Renal Sympathetic Denervation

Renal sympathetic denervation (RSD) by ablation catheter reduces renal efferent activity and, consequently, increases renal blood flow and decreases activation of the renin-angiotensin-aldosterone system, water retention, and renal afferent activity, which through brain signaling, decreases sympathetic action on heart and vessels.^160^

Data from uncontrolled studies have shown reductions of up to 30 mmHg in office systolic BP in patients with RHTN, without complications related to the procedure.^161^However, the SYMPLICITY HTN-3 trial,^162^a randomized sham-controlled study, showed no significantly superior effect of BP reduction after 6 months from RSD. A meta-analysis of 11 controlled studies comparing RSD with optimized pharmacological treatment or sham procedure in patients with RHTN showed that RSD was not superior in reducing BP, with heterogeneity of responses in the studies, mainly due to lack of a sham control in most publications and heterogeneity in assessment of treatment adherence.^163^

The development of new circumferential catheters with distal renal artery applications may promote a more complete RSD, and their effects on BP reduction have been demonstrated in patients with untreated hypertension.^164^

The 2018 European Society of Hypertension position paper does not recommend RSD for treatment of hypertension in general but includes a recommendation of this procedure in the context of controlled clinical studies with sham procedures and optimized therapy for safety and efficacy assessment in populations with a large number of individuals.^160^

Based on this evidence, RSD is currently an alternative only for patients with UC-RHTN with optimized pharmacological treatment and proven therapeutic adherence or with important drug-related adverse effects, to be always performed at referral centers trained for the procedure.^164^

### 10.4. Use of Continuous Positive Airway Pressure

OSA is a clinical condition affecting more than half of the patients with RHTN^94^and is mainly treated with CPAP, an air compressor that applies continuous positive pressure to the patient’s airway. To date, seven randomized trials have analyzed the effect of treatment of OSA with CPAP in patients with RHTN.^165 - 171^Except for one of these studies,^170^the others found significant reductions in BP (5 mmHg on average; one study showed reductions ≥ 10 mmHg after CPAP use).^169^

However, the proportion of patients who achieved the target BP (< 140/90 mmHg) with CPAP was low, possibly due to poor CPAP adherence. In clinical practice, the BP response to CPAP varies, even in patients with good adherence. A recent study showed predictive biomarkers of better BP response to CPAP in patients with RHTN.^172^Validation and large-scale application of these biomarkers could help select better those patients who benefit most from BP reduction.

### 10.5. Arteriovenous Fistula

The creation of an arteriovenous fistula (AVF) can decrease BP by reducing total peripheral resistance and blood volume and inducing baroreflex inhibition and release of natriuretic peptides.^173^In a prospective randomized controlled trial, the creation of a central iliac AVF by an implantable device in 44 patients with RHTN led to significant reductions in 24-hour office and ambulatory systolic BP compared with pharmacological treatment.^174^However, there was a high rate of complications due to ipsilateral venous stenosis, requiring intervention in the AVF group.

Further studies with a greater number of patients and with a comparison of AVF versus sham procedure are being conducted to verify the benefits of AVF in RHTN.^173^
